# Auditory Sensitivity in Autism: A Systematic Review of Mismatch Negativity and Mismatch Field Responses

**DOI:** 10.1002/aur.70275

**Published:** 2026-05-17

**Authors:** Sara Cacciato‐Salcedo, Shreya Jaokar, Neil J. Ingham, Manuel S. Malmierca, Marija M. Petrinovic

**Affiliations:** ^1^ Cognitive and Auditory Neuroscience Laboratory (CANELAB) Institute of Neuroscience of Castilla and Leon (INCYL), Calle Pintor Fernando Gallego Salamanca Spain; ^2^ Department of Forensic and Neurodevelopmental Sciences Institute of Psychiatry, Psychology and Neuroscience, King's College London London UK; ^3^ Wolfson Sensory, Pain and Regeneration Centre King's College London London UK; ^4^ Institute for Biomedical Research of Salamanca (IBSAL) University of Salamanca Salamanca Spain; ^5^ Department of Cell Biology and Pathology, Faculty of Medicine University of Salamanca Salamanca Spain; ^6^ Department of Neuroimaging Institute of Psychiatry, Institute of Psychiatry, Psychology and Neuroscience, King's College London London UK; ^7^ Medical Research Council Centre for Neurodevelopmental Disorders King's College London, New Hunt's House London UK

**Keywords:** autism spectrum disorder (ASD), electroencephalography (EEG), magnetoencephalography (MEG), mismatch fields (MMF), mismatch negativity (MMN)

## Abstract

Auditory mismatch responses—mismatch negativity (MMN) and mismatch fields (MMF)—are well established electrophysiological markers of automatic auditory discrimination supported by short‐term sensory memory. These responses, typically elicited using passive oddball paradigms, are increasingly used to investigate sensory and language processing in autism. This systematic review synthesizes findings from 55 studies comparing MMN and MMF responses in autistic and typically developing (TD) individuals across childhood, adolescence, and adulthood. Using the Synthesis Without Meta‐analysis (SWiM) framework, we identified consistent evidence for smaller MMN amplitudes and reduced MMF power in autistic children and adolescents relative to TD peers, particularly in response to frequency, duration, and speech‐based deviants. Studies also frequently reported longer mismatch latencies in autistic participants and associated these delays with language difficulties and heightened auditory sensitivity. Although some studies reported age‐related convergence in MMN and MMF measures between autistic and TD groups in later childhood or adolescence, greater right‐hemisphere lateralization in autistic individuals emerged as a consistent finding across both speech and non‐speech paradigms, suggesting differences in hemispheric weighting for auditory processing of linguistic and non‐linguistic cues. To explain interindividual and developmental variability in mismatch responses, we propose a precision‐weighted predictive coding account, in which divergent assignment of confidence to sensory prediction errors may contribute to autism‐related differences. While study quality was generally fair, methodological heterogeneity, underrepresentation of females, and limited cross‐cultural sampling constrain generalizability. Future research should prioritize longitudinal, sex‐stratified, and culturally diverse designs, using standardized protocols and collaborative data practices. MMN and MMF responses hold promise as non‐invasive translational biomarkers of early‐stage sensory prediction and neurodevelopmental variation in autism.

## Summary

Autistic people often experience sounds differently from the typically developing (TD) population. We reviewed 55 studies of two brain responses—mismatch negativity (MMN) and mismatch fields (MMF)—that reflect how the brain automatically detects changes in sound patterns without requiring active attention.

Across children and adolescents, autistic groups commonly showed smaller and delayed MMN and MMF than TD groups to changes in tone, duration, and speech sounds, along with greater right‐hemisphere involvement. These patterns were sometimes linked to language development and heightened sound sensitivity. Findings in autistic adults were more mixed, suggesting that some differences from TD peers may lessen with age but can persist depending on the type of sound and task demands.

Overall, MMN and MMF provide valuable non‐invasive indices of early auditory prediction and discrimination processes in autism. However, studies used varied methods, included fewer girls and women, and were conducted in a limited range of regions. We recommend larger, more diverse, and longitudinal studies using consistent protocols to clarify how these neural responses relate to everyday listening and language.

## 1 Introduction

From early infancy onward, the brain extracts regularities from the acoustic environment to support the maturation of speech perception, language learning, and social communication. This capacity depends on automatic, largely pre‐attentive mechanisms that form short‐lived representations of repeating sound patterns and generate mismatch responses when incoming input deviates from established regularities. Across development, the stability and fidelity of these regularity representations shape how efficiently deviations are detected within complex auditory environments.

In autism, auditory regularity formation and deviance detection may follow a different and more variable developmental course than in TD peers (Gomot et al. 2006; Haesen et al. 2011; Marco et al. 2011). Autism is a neurodevelopmental condition characterized by early emerging differences in social communication alongside restricted and repetitive patterns of behavior (American Psychiatric Association (APA) 2022). Many autistic individuals report heightened or diminished responsiveness to auditory input, spanning both social and non‐social sounds (Glod et al. 2015; Gomes et al. 2008; Ha et al. 2015). These sensory differences often emerge early and may influence how reliably the auditory system forms and maintains regularities in everyday environments, with downstream implications for language development and participation across contexts (Eberhardt and Nadig 2018; Haesen et al. 2011; Poulsen et al. 2024). As communicative demands increase across the lifespan, many autistic individuals encounter growing social and academic challenges, reflecting interacting sensory, cognitive, and environmental influences rather than a single underlying cause (Levy and Perry 2011; Magiati et al. 2014; Ploog et al. 2013; Whitehouse et al. 2009).

During typical development, infants and children progressively extract linguistically meaningful units such as phonemes, which scaffold later language learning (Atienza et al. 2002; Cheour et al. 1998; Mueller et al. 2012). Auditory sensory‐memory mechanisms support this process by sustaining short‐lived representations of the recent acoustic context and enabling comparisons between incoming input and established regularities. When a discrepancy arises, automatic change‐detection mechanisms engage and generate mismatch responses (Garrido et al. 2009; Näätänen et al. 2001, 2007, 2011). Over time, the auditory system increasingly tunes to language‐relevant features, supporting efficient detection of subtle deviations embedded within complex soundscapes (Näätänen et al. 2011; Näätänen and Alho 1997; Vouloumanos and Werker 2004). Divergences in the formation or maintenance of these regularities may therefore influence how reliably deviations are detected in autism.

Auditory mismatch responses are most commonly measured as mismatch negativity (MMN) using electroencephalography (EEG) or as mismatch fields (MMF) using magnetoencephalography (MEG). The literature generally treats MMN and MMF as closely related indices of automatic auditory deviance detection and the integrity of short‐term representations of auditory regularities (Atienza et al. 2002; Foss‐Feig et al. 2012; Haesen et al. 2011; Kujala et al. 2013; Näätänen et al. 2012). These responses are typically elicited using passive auditory oddball paradigms, in which infrequent deviants (e.g., frequency, duration, intensity, or speech‐feature changes) violate regularities established by repeated standards (Garrido et al. 2009; Kujala et al. 2013; Näätänen et al. 2011). Larger MMN amplitudes or stronger MMF responses are commonly interpreted as reflecting a clearer neural contrast between deviant input and the established regularity, whereas smaller responses are interpreted as reflecting weaker or less consistently expressed mismatch signaling under a given stimulus context (Foss‐Feig et al. 2012; Haesen et al. 2011; Näätänen et al. 2011).

Mismatch responses are also interpreted within predictive‐processing frameworks, which describe perception as an inferential process in which internal expectations are updated when sensory input deviates from predicted patterns (Friston 2005; Garrido et al. 2009). Within this framework, MMN and MMF are commonly viewed as neural correlates of the prediction error elicited when an established regularity is violated (Näätänen et al. 2012; Orekhova and Stroganova 2014). Rather than representing mutually exclusive explanations, predictive and sensory‐memory accounts converge in proposing that reliable deviance detection depends on the stability and fidelity of inferred regularity representations (Kujala et al. 2013). MMN and MMF therefore likely reflect interacting influences from regularity formation, sensory memory, neural adaptation, and predictive updating rather than a single mechanism in isolation.

Across typical development, mismatch responses continue to mature from childhood through adolescence, although reported trajectories vary across studies and depend on stimulus features and task design. Some studies report age‐related increases in MMN amplitude, whereas others describe decreases or minimal change, highlighting the sensitivity of these measures to paradigm characteristics (Bishop et al. 2011; Chow et al. 2025; Gaeta et al. 1998; Lindín et al. 2013). Speech‐evoked paradigms also yield heterogeneous findings, with some studies reporting age‐related differences in MMN latency under specific stimulus contexts (Chobert et al. 2014; Cooper et al. 2006; Csépe 1995; Kraus et al. 1993; Linnavalli et al. 2018). Together, these results suggest prolonged maturation of regularity representations and deviance detection across development, with trajectories shaped by task demands, stimulus properties, and participant characteristics.

In autism, developmental trajectories of regularity formation and deviance detection may diverge from those observed in TD peers. Differences in the formation and maintenance of regularity representations, as well as in the efficiency with which deviations are registered, may contribute to variability in the perception of dynamic auditory environments across childhood, adolescence, and adulthood, with potential implications for language‐ and communication‐relevant auditory computations (Green et al. 2020; O'Connor 2012; Rapaport and Sowman 2024). MMN and MMF are well suited to examine these questions because they do not require an overt behavioral response and are less sensitive to task demands that may differ between autistic individuals and TD peers in active discrimination paradigms (Chen et al. 2020; Haesen et al. 2011; Kujala et al. 2013).

This review therefore focuses on passive oddball paradigms because they target automatic regularity formation and deviance detection under minimal task demands and reduce confounds that may differ systematically between autistic individuals and TD peers. Passive designs limit the influence of instruction comprehension, strategic responding, sustained attention, and motor requirements, which can otherwise obscure whether observed group differences reflect auditory regularity processing or task‐related factors (Foss‐Feig et al. 2012; Garrido et al. 2009; Kikuchi et al. 2016; Orekhova and Stroganova 2014). By contrast, active paradigms more directly probe how attention, decision processes, and task goals shape deviance detection, and differences between passive and active findings may therefore reflect contextual influences rather than simple inconsistency (Kujala et al. 2013). Despite these advantages, passive oddball studies in autism report heterogeneous MMN/MMF amplitudes, latencies, and spatial distributions across ages, stimulus classes, and participant profiles (Chen et al. 2020; Haesen et al. 2011; Schwartz et al. 2018; Seri et al. 2007). The literature has also rarely tested and has not yet resolved how mismatch characteristics relate to language development and social communication (Kikuchi et al. 2016).

A recent systematic review and meta‐analysis (Sapey‐Triomphe et al. 2025) quantified group differences in auditory MMN amplitude and latency in autism and examined developmental and paradigm‐related moderators within a predictive coding framework. That study provided pooled effect size estimates and demonstrated that MMN amplitude differences varied as a function of age group and design characteristics, with reduced amplitudes most consistently observed in autistic children and adolescents, and some multi‐feature paradigms in adults showing the opposite pattern relative to TD peers. Latency effects were less consistent. However, the quantitative synthesis was restricted to EEG‐based MMN studies and focused on pooled amplitude and latency estimates, necessarily excluding MEG investigations and studies that lacked extractable summary statistics.

The present review complements and extends that work. First, we integrate findings from both EEG (MMN) and MEG (MMF) studies, enabling cross‐modal comparison of amplitude, latency, and spatial characteristics, including hemispheric distribution—an aspect not addressed in prior quantitative syntheses. Second, instead of re‐estimating pooled EEG effect sizes, we apply a Synthesis Without Meta‐analysis (SWiM) framework to accommodate substantial methodological and clinical heterogeneity across paradigms, stimulus classes, electrode montages, and participant phenotypes (Campbell et al. 2020). This approach allows us to retain mechanistically informative studies that are not meta‐analyzable and to examine boundary conditions that may not be visible in pooled effect‐size models. Third, we systematically evaluate hemispheric weighting, speech versus non‐speech contrasts, and phenotype‐linked moderators such as language ability and auditory sensitivity—domains that were not central outcomes of previous meta‐analyses.

By emphasizing spatial organization, developmental modulation across recording modalities, and functional correlates of mismatch responses—alongside group‐level effects—this review provides a complementary and mechanistically oriented perspective on auditory regularity formation and deviance detection in autism. Rather than focusing solely on the magnitude or direction of pooled EEG group differences, we examine when, where, and under which phenotypic conditions mismatch responses diverge.

Accordingly, this systematic review synthesizes studies measuring auditory MMN and MMF in autistic individuals and TD peers across development, focusing on passive oddball paradigms using speech and non‐speech sounds. We aim to (1) characterize developmental variation in mismatch responses in autism; and (2) evaluate how MMN/MMF characteristics—particularly amplitude, latency, and when reported, spatial distribution—relate to language and social‐communication‐relevant measures, while identifying methodological and participant‐level moderators that plausibly explain cross‐study heterogeneity.

By clarifying the functional significance and developmental modulation of mismatch responses in autism, this review seeks to strengthen interpretation of auditory deviance‐detection findings and to guide more comparable, phenotype‐informed study designs in future work.

## 2 Methods

### 2.1 Protocol and Registration

The protocol for this systematic review was registered in the International Prospective Register of Systematic Reviews (PROSPERO; registration number: CRD42025626806). The review was conducted in accordance with the Preferred Reporting Items for Systematic Reviews and Meta‐Analyses (PRISMA) guidelines (Page et al. 2021).

### 2.2 Search Strategy and Study Selection

**FIGURE 1:**
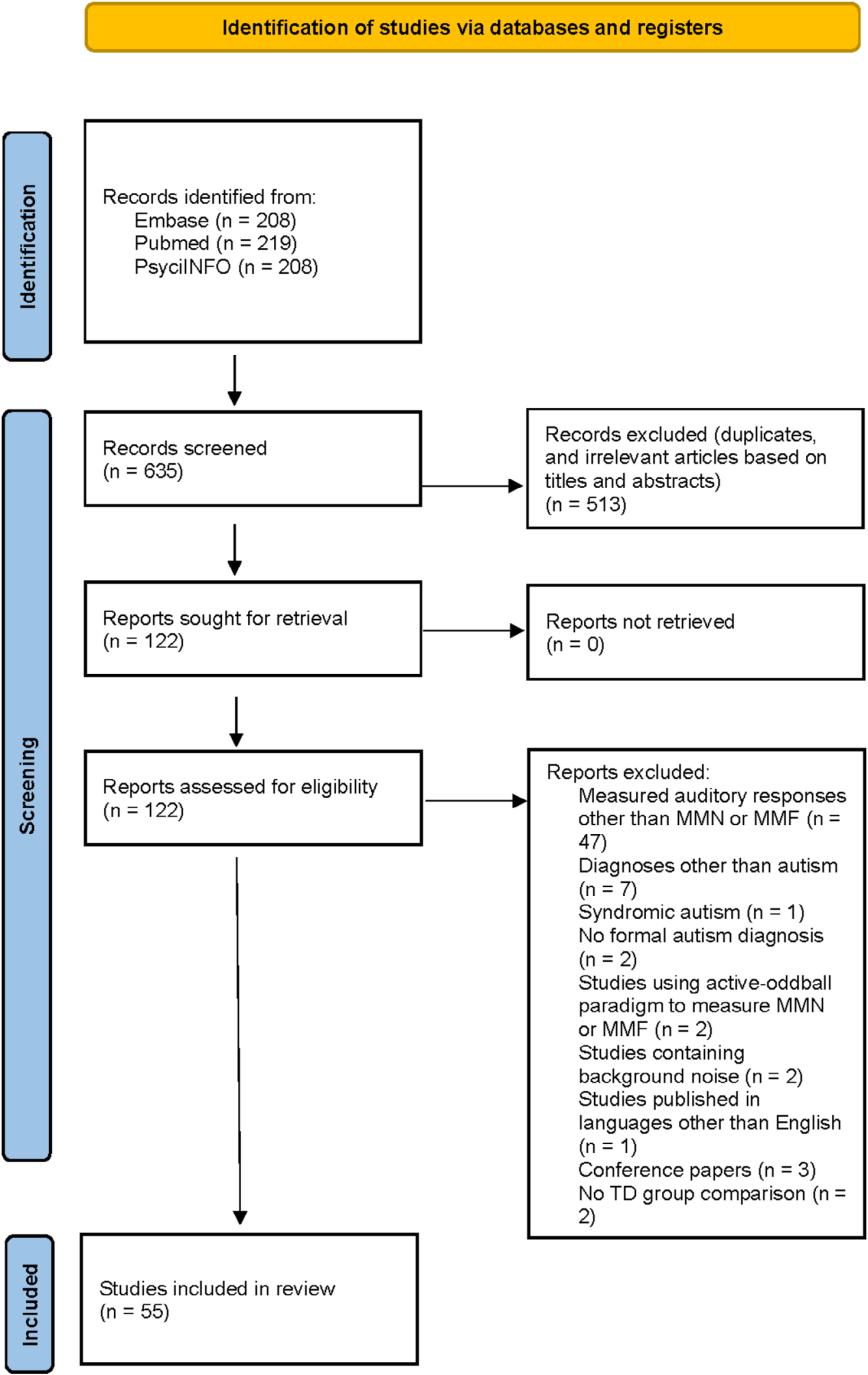
PRISMA flow diagram. MMF, mismatch field; MMN, mismatch negativity; TD, typically developing.

We conducted a systematic search of the Embase, Medline, and PsycInfo databases from their inception to 31 May 2025, using the strategy outlined in Supporting information. Additional eligible studies were identified by screening the reference lists of included articles. The search and study selection processes are detailed in the PRISMA flow diagram (Figure 1).

Studies were included if they met the following criteria: (1) reported original research; (2) investigated auditory MMN or MMF amplitude or power, respectively, and latency; (3) employed passive oddball paradigms; (4) used speech or non‐speech auditory stimuli; (5) compared autistic and TD individuals; (6) included children, adolescents, or adults; and (7) excluded individuals with hearing difficulties.

No restrictions were placed on year of publication, or the age or sex/gender of participants. However, included studies were required to involve participants with a formal diagnosis of autism based on the DSM‐III (APA 1980), DSM‐IV (APA 1994), DSM‐IV‐TR (APA 2000), DSM‐5 (APA 2013), International Statistical Classification of Diseases Tenth Revision (ICD‐10) (World Health Organization (WHO) 2016), or ICD‐11 (WHO 2019). As the DSM‐5 and ICD‐11 consolidate Asperger's syndrome and other pervasive developmental disorders (PDDs) under the broader category of autism spectrum disorder (ASD), we refer to this group collectively as “autistic” throughout the review. For transparency, original diagnostic labels used in each study are reported in Tables 1, 2, 3, 4, 5, 6.

**TABLE 1:** Demographic and methodological data from MMN and MMF studies in children.

Study	Diagnosis	Cases (*n*)	TD (*n*)	Mean age in years, cases (TD)	Sex, male (female)	Response and neuroimaging technique	Recording sites: MMN electrodes/MMF regions	Background activity
Abdeltawwab and Baz (2015)	Autism	31	30	11.3 (11.2)	42 (19)	MMN using EEG	Fz, A1, A2	Pictured book
Berman et al. (2016)	ASD	95	44	10.2 (10.4)	Not reported	MMF using 275‐channel MEG	STG	Not specified
Cary et al. (2024)	ASD	13	13	12.8 (12.5)	ASD: 11 (2) TD: 6 (7)	MMN using EEG	FCz (128‐channel EGI system)	Silent movie
Čeponienė et al. (2003)	Autistic disorder	9	10	8.9 (8.4)	17 (2)	MMN using EEG	F3, F4, C3, C4	Silent video
Charpentier et al. (2018)	ASD	Children (15), Adults (16)	Children (15), Adults (16)	Children: 10 (9.8); adults: 26.2 (26.2)	Children: 25 (5); adults: 28 (4)	MMN using EEG	Children: Cz, CPz, Pz; adults: Fz, FCz, Cz, CPz, Pz	Silent video
Da Silva Mayerle et al. (2023)	ASD	17	51	7–17 (7–17)	ASD: 15 (2) TD: 45 (6)	MMN using EEG	Fz, M1, M2	Silent video on tablet
Di Lorenzo et al. (2020)	ASD; ASD + APS	ASD = 21; ASD + APS = 16	20	13.67 (13.04)	ASD + ASD + APS: 32 (8); TD: 12 (8)	MMN using EEG (64‐channel)	Fz (MMN quantified); 61 scalp electrodes; M1/M2 mastoids	Silent cartoon video
Ferri et al. (2003)	Autistic disorder	10	10	12.3 (12.2)	20 (0)	MMN using EEG	Fz, Cz, Pz	Silent video
Gomot et al. (2002)	Infantile autism	15	15	6.1 (6.9)	24 (6)	MMN using EEG	Fz, M1, M2	Silent video
Gomot et al. (2011)	Autism	27	27	8.4 (8.4)	42 (12)	MMN using EEG	Fz, Cz, Pz, T3, T4, M1, M2	Silent video
Green et al. (2020)	ASD with and without language impairment (ASD + LI; ASD–LI)	15	10	7.3 (8.5)	ASD: 12 (3); TD: 7 (3)	MMN using EEG	Frontocentral electrodes (left and right montages; 128‐channel EGI system)	Silent movie
Huang et al. (2018)	Autistic disorder	22 18	20 17	9.6 (9.4) 9.8 (9.4)	36 (6) 31 (4)	MMN using EEG	M1	Silent video
Jansson‐Verkasalo et al. (2003)	AS	10	11	9.1 (9.1)	13 (8)	MMN using EEG	F4, C4, P4, T4, Fz, Cz, Pz, Oz, F3, C3, P3, T3	Silent video
Jansson‐Verkasalo et al. (2005)	AS	Children (19); fathers (15); Mothers (19)	Children (18); fathers (14); mothers (17)	Children: 10.6 (10.4)	23 (14)	MMN using EEG	F4, C4, P4, T4, Fz, Cz, Pz, Oz, F3, C3, P3, T3	Silent video
Kabil et al. (2023)	ASD	60	60	9.8 (10.3)	ASD: 61.7% male TD: 55% male	MMN using EEG	Not specified; MMN evoked during silent video viewing	Silent video
Kemner et al. (1995)	Infantile autism	20	TD peers (20); ADHD‐HI (20); dyslexia (20)	Autism (9.8); TD peers (10.6); ADHD‐HI (9.9); dyslexia (10)	Not reported	MMN using EEG	F3, Fz, F4, C3, Cz, C4, P3, Pz, P4, O1, Oz, O2	Visual fixation point
Korpilahti et al. (2007)	AS	Boys (14); fathers (12)	Boys (13); fathers (13)	Boys: 11.2 (10.8); fathers: 42.8 (44.3)	Boys: 27; fathers (25)	MMN using EEG	F4, C4, CP6, P4, Fz, Cz, Pz, F3, C3, CP5, P3	Silent video
Kujala et al. (2010)	AS	15	13	10.9 (10.6)	22 (6)	MMN using EEG	Fz, Cz	Silent video
Lassen et al. (2022)	ASD	59	59	11.89 (11.75)	ASD: 49 (10) TD: 47 (12)	MMN using 64‐channel BioSemi EEG	Fz, FCz, Cz	Watched muted cartoon movie during MMN task
Lepistö et al. (2005)	Autistic disorder	15	15	9.4 (9.4)	26 (4)	MMN using EEG	F3, C3, T3, TP3, Fz, Cz, Pz, F4, T4, P4, TP4	Silent video
Lepistö et al. (2006)	AS	10	10	8.11 (8.10)	16 (4)	MMN using EEG	F3, C3, T3, TP3, Fz, Cz, Pz, F4, T4, P4, TP4	Silent video
Lepistö et al. (2008)	Autism	10	10	9.1 (9)	18 (2)	MMN using EEG	F3, F4, Fz, C3, C4, Cz, TP3, TP4, Cz	Silent video
Lepistö et al. (2009)	AS	16	14	8.1 (8.1)	25 (5)	MMN using EEG	Fcz, F3, F4, Fc3, Fc4, C3, C4, Cp3, Cp4, P3, P4	Silent video
Lindström et al. (2016)	Autism	10	13	10.5 (10)	21 (2)	MMN using EEG	Cz	Silent video
Lindström et al. (2018)	ASD	ASD (2); AS (13)	16	10.4 (10.1)	31 (0)	MMN using EEG	Pz	Silent video
Lortie et al. (2017)	ASD	10	12	6.71 (6.72)	17 (5)	MMN using EEG	FCz	Silent video
Matsuba et al. (2024)	ASD	10	21	12.7 (12.8)	ASD: 91% male TD: 43% male	MMN using 128‐channel EEG	Frontocentral (E5, E6, E7, E12, E106), Left (E39, E40, E45, E46, E50), Right (E101, E102, E108, E109, E115)	Silent movie/TV show
Matsuzaki et al. (2017)	ASD	20	13	9.62 (9.45)	33 (0)	MMF using 160‐channel MEG system	Temporal and frontal regions	Visual fixation point
Oram Cardy et al. (2005)	Autistic disorder	7	9	11.9 (11.9)	12 (4)	MMF using 151‐channel MEG system	Left hemisphere	Silent video
Roberts et al. (2011)	ASD	51	27	8.47 (10)	61 (17)	MMF using 275‐channel MEG	STG	Silent video
Ruiz‐Martinez et al. (2020)	ASD	16	15	8.96 (8.86)	29 (2)	MMN using EEG	Fz, F3, F4	Silent video
Sanglakh Ghoochan Atigh et al. (2024)	ASD	20	24	4.5–11 (4.5–11)	80% male (ASD), 71% male (TD)	MMN using 19‐channel EEG	Fz, Cz	Silent video
Sawada et al. (2008)	PDD	10	0	8.6	10 (0)	MMN using EEG	Fz, Cz, Pz	Reading a book
Schall et al. (2024)	ASD	27	26	8.89 (10.09)	ASD: 21 (6) TD: 15 (11)	MMN using EEG (32‐channel Quick‐Cap)	Fz, F3, F4, Cz, C3, C4, Pz, P3, P4, M1, M2	Silent movie
Vlaskamp et al. (2017)	Autistic disorder, AS or PDD‐NOS	Autistic disorder (11); AS (7); PDD NOS (17)	38	11.1 (10.9)	55 (18)	MMN using EEG	Fz, FCz, Cz	Viewing written instructions to sit still
Weismüller et al. (2015)	ASD	18	15	9.4 (10.6)	33 (0)	MMN using EEG	Fz, F3, F4, Cz, C3, C4, Pz, P3, P4, M1, M2	Silent video
Yu et al. (2015)	Autistic disorder	17 16	15 18	9.3 (9.5) 9.3 (9.6)	27 (5) 29 (5)	MMN using EEG	Fz, Cz	Silent video
Zhang et al. (2018)	Autism, AS, PDD‐NOS	15	16	10.04 (9.48)	22 (9)	MMN using EEG	CP3, CP4, T7, T8, P7, P8, TP9, TP10	Silent video
Zhang et al. (2019)	Autism, Autistic disorder, AS, PDD‐NOS	16	16	10.42 (9.48)	23 (9)	MMN using EEG	Fz	Silent video

Abbreviations: ADHD, attention deficit hyperactivity disorder; APS, attenuated psychosis syndrome; AS, Asperger's syndrome; ASD, autism spectrum disorder; EEG, electroencephalography; LI, language impairment; MEG, magnetoencephalography; MMF, mismatch field; MMN, mismatch negativity; PDD, pervasive developmental disorder; PDD‐NOS, pervasive developmental disorder‐not otherwise specified; TD, typically developing.

**TABLE 2:** Demographic and methodological data from MMN and MMF studies in adolescents.

Study	Diagnosis	Cases (*n*)	TD (*n*)	Mean age in years, cases (TD)	Sex, male (female)	Response and neuroimaging technique	Recording sites: MMN electrodes/MMF regions	Background activity
Da Silva Mayerle et al. (2023)	ASD	7	13	13.9 (14.0)	11 (9)	MMN using EEG	Fz, M1, M2	Silent video on tablet
Hudac et al. (2018)	ASD	102	31	12.29 (13.27)	103 (30)	MMN using EEG	Central medial electrodes	Silent video
Lassen et al. (2022)	ASD	59	59	11.89 (11.75)	49 (10)	MMN using EEG	Fz, FCz, Cz	Muted cartoon movie
Ludlow et al. (2014)	Autistic disorder or AS	HFA (9); AS (2)	11	13 (13.7)	22 (0)	MMN using EEG	CPz	Silent video
Mamashli et al. (2017)	ASD	19	17	12 (13)	36 (0)	MMF using whole‐head VectorView MEG system	STG, MTG, IFG	Silent video
Sokhadze et al. (2016)	Autism	16	3	12.6 (12.6)	15 (3)	MMN using EEG	Fz, FC1, FC2, Cz	Viewing written instructions to sit still
Tecchio et al. (2003)	Autistic disorder	14	10	16 (19)	19 (5)	MMF using 28‐channel MEG system	Midtemporal lobe contralateral to the stimulated ear	Not specified

Abbreviations: AS, Asperger's syndrome; ASD, autism spectrum disorder; CPz, centroparietal midline electrode; Cz, central midline electrode; EEG, electroencephalography; FCz, frontocentral midline electrode; Fz, frontal midline electrode; HFA, high‐functioning autism; IFG, inferior frontal gyrus; M1/M2, left/right mastoid electrode; MEG, magnetoencephalography; MMF, mismatch field; MMN, mismatch negativity; MTG, middle temporal gyrus; STG, superior temporal gyrus.

**TABLE 3:** Demographic and methodological data from MMN and MMF studies in adults.

Study	Diagnosis	Cases (*n*)	TD (*n*)	Mean age in years, cases (TD)	Sex, male (female)	Response and neuroimaging technique	Recording sites: MMN electrodes/MMF regions	Background activity
Chien et al. (2018)	Autistic disorder, AS	Autistic disorder (11), AS (26)	35	21 (20.5)	67 (5)	MMN using EEG	Fz	Silent video
Fan and Cheng (2014)	AS and HFA	20	20	21.5 (22)	38 (2)	MMN using EEG	Fz, Cz, Pz	Silent video
Goris et al. (2018)	ASD, autistic disorder, or AS	18	24	21 (21.2)	29 (14)	MMN using EEG	F3, Fz, F4, FC3, FCz, FC4, C3, Cz, C4	Silent video
Haigh et al. (2023)	ASD	21	25	29.5 (33.6)	32 (14)	EEG (MMN, TTV)	F1, Fz, F2, FC1, FCz, FC2, C1, Cz, C2	Visual fixation‐cross task
Kasai et al. (2005)	Autistic disorder	9	19	27.2 (27.3)	19 (9)	MMF using 122‐channel MEG	Temporal region (bilateral)	Silent video
Kujala et al. (2005)	AS	8	8	33 (32)	8 (8)	MMN using EEG	Fz, F3, CP3, CT3, TP3, F4, CP4, CT4, TP4, Cz	Silent video
Kujala et al. (2007)	AS	8	10	27 (30)	14 (4)	MMN using EEG	Fz, F3, F4, Cz, C3, C4, Pt3, Pt4, T3, T4, M1, M2	Silent video
Lepistö et al. (2007)	AS	9	9	27 (30)	15 (3)	MMN using EEG	F3, C3, T3, TP3, Fz, Cz, Pz, F4, T4, P4, TP4	Silent video
Matsuzaki et al. (2019)	ASD	9	16	22.2 (27.3)	25 (0)	MMF using 275‐channel MEG	STG	Silent video
Randeniya et al. (2022)	ASD	23	23	24.35 (24.04)	ASD: 10 (12)1 intersex TD: 11 (12)	MMN using EEG	Fz; whole‐scalp (64‐ch)	Visual 2‐back task
Sato et al. (2025)	ASD	15	20	29.7 (23.9)	20 (15)	MMN using EEG	Fz, Cz	Silent movie

*Note:* Electrodes mentioned include Fz (frontal midline), Cz (central midline), Pz (parietal midline), FCz (frontocentral midline), CPz (centroparietal midline), Oz (occipital midline), and several lateral sites such as F3, F4, C3, C4, P3, P4, T3, T4, TP3, TP4, A1, A2, M1, and M2, corresponding to left/right frontal, central, parietal, temporal, temporoparietal, auricular, and mastoid locations based on the international 10–20 system.

Abbreviations: AS, Asperger syndrome; ASD, autism spectrum disorder; EEG, electroencephalography; HFA, high functioning autism; MEG, magnetoencephalography; MMF, mismatch field; MMN, mismatch negativity; SPM, statistical parametric mapping software used in neuroimaging analysis; STG, superior temporal gyrus, TD, typically developing; TTV, trial‐to‐trial variability.

**TABLE 4:** Auditory MMN and MMF responses in children.

Stimuli characteristics				Study findings						
Study	Standard stimulus	Deviant stimulus	Difference between stimuli	Enhanced MMN amplitude or MMF power in ASD cases compared to TD peers	Attenuated MMN amplitude or MMF power in ASD cases compared to TD peers	No between‐group difference in MMN amplitude or MMF power	Earlier MMN or MMF latency in ASD cases compared to TD peers	Prolonged MMN or MMF latency in ASD cases compared to TD peers	No between‐group difference in MMN and MMF latencies	Other findings
Abdeltawwab and Baz (2015)	1000 Hz tone bursts	1500 Hz tone bursts	Sound frequency	−	*p* < 0.05	−	−	−	+	MMN amplitude positively correlated with age in ASD.
Berman et al. (2016)	Vowel /a/	Vowel /u/	Phoneme change	−	−	−	−	−	−	ASD + LI group displayed prolonged latencies (~25 ms later) than ASD‐LI (*p* < 0.02).
Cary et al. (2024)	1000 Hz pure tone	1200 Hz pure tone	Sound frequency	−	−	+	−	−	+	Reduced P1 habituation in ASD; MMN amplitude correlated with sensory overresponsivity and autistic traits; no group differences in MMN amplitude or latency
Čeponienė et al. (2003)	Simple tone 458 Hz; Complex tone‐combination of 458, 1370, 2054, and 3537 Hz sinusoidal tones; Finnish vowel /o/	Increased frequency by 10% of each standard stimulus	Sound frequency	−	−	+	−	−	+	Larger complex‐tone MMN amplitude than simple‐tone MMN amplitude (*p* < 0.001) in both groups
Charpentier et al. (2018)	/a/ in neutral prosody for 400 ms at 70% dB SPL	/a/ uttered with anger	Emotional prosody	−	*p* = 0.029	Only adult participants	*p* = 0.041	−	−	The preference for knowledge and the neural representation of prediction errors in gaining knowledge are valence‐dependent
Da Silva Mayerle et al. (2023)	1000 Hz tone burst	2000 Hz tone burst	Sound frequency	−	*p* < 0.001 (children group)	−	−	*p* < 0.001 (children group)	−	High auditory hypersensitivity; altered morphology in MMN waveforms; different auditory processing in ASD.
Di Lorenzo et al. (2020)	1000 Hz tone, 50 ms	1200 Hz tone, 50 ms; 1000 Hz tone, 100 ms	Sound frequency; sound duration	−	Reduced MMN amplitude for frequency and duration deviants in ASD vs. TD (*p* < 0.01)	−	−	Prolonged MMN latency for frequency deviants in ASD vs. TD (*p* = 0.006)	Duration‐MMN latency: no group difference	MMN latency negatively correlated with ADOS‐2 severity in ASD + APS for frequency (r = −0.810, *p* < 0.0001) and duration (r = −0.650, *p* = 0.006); APS status did not affect MMN indices
Ferri et al. (2003)	1000 Hz sinusoidal tones	Deviant: 1300 Hz sinusoidal tones; novel: complex and non‐monotonal tones with same duration but new spectral content	Sound frequency and spectral content	*p* < 0.041 at Cz electrode for deviant‐standard condition	−	Novel‐standard condition	−	−	Novel‐standard condition	In ASD at 8 years, the regression line for MMN amplitude over Fz and Cz was higher than in TD peers but grew closer to the latter by 18 years.
Gomot et al. (2002)	1000 Hz	1100 Hz	Sound frequency	−	−	−	Fz (*p* < 0.04), M1 (*p* < 0.03), M2 (*p* < 0.02) electrodes	−	−	Differences in MMN generators in ASD (C3, C4 electrodes) and TD peers (Fz).
Gomot et al. (2011)	1000 Hz	1100 Hz	Sound frequency	−	M2 electrode (*p* = 0.003)	−	−	−	−	Shorter MMN latencies at M1 (*p* = 0.011) and M2 (*p* = 0.027) for ASD participants who displayed high intolerance to change.
Green et al. (2020)	300 Hz pure tone; vowel /ɑ/	700 Hz pure tone; vowel /u/	Sound frequency; phoneme identity	−	−	−	− Left frontocentral montage (*p* = 0.021)	−	−	In the vowel sound condition, earlier MMN latency to vowel deviants in ASD + LI compared to ASD–LI and TD
Huang et al. (2018)	295 Hz pure tone for 250 ms; Mandarin non‐sense syllable /du/ for 250 ms	295 Hz pure tones for 350 ms; lengthened the vowel portion of /du/ to 350 ms	Duration	− −	*p* < 0.05 −	− +	− −	*p* < 0.05 −	− +	No additional findings
Jansson‐Verkasalo et al. (2003)	1000 Hz tone; Finnish syllable /taa/	1100 Hz tone; Finnish syllable /kaa/	Sound frequency Phoneme change	− −	− −	+ +	− −	Right (*p* < 0.00002) and left (*p* < 0.0008) hemispheres Right hemisphere (*p* < 0.002)	−	Larger right than left hemisphere MMN in AS but opposite in TD peers
Jansson‐Verkasalo et al. (2005)	Finnish word “anna” at 280 Hz	Finnish word “anna” at 320 Hz	Sound frequency	−	Frontal (*p* = 0.0000) and central (*p* = 0.025) regions in children	−	−	In AS children (*p* = 0.003) and fathers of AS children compared to fathers of TD children (*p* = 0.029)	Early‐MMN latency in AS children	Early‐MMN in left hemisphere (*p* = 0.002) and at C3 (*p* = 0.040), P3 (*p* = 0.040), 44 (*p* = 0.040) electrodes in fathers of AS children than those of TD peers
Kabil et al. (2023)	1000 Hz tone burst	2000 Hz tone burst	Sound frequency	−	−	+	−	Prolonged MMN latencies in ASD vs. TD peers (*p* = 0.035 RE, *p* = 0.005 LE)	−	30% ASD cases showed MMN latency differences compared to TD peers
Kemner et al. (1995)	Phoneme /oy/	Deviant /ay/; novel /bbrrzzz/ sound	Phoneme change	−	−	+	−	−	+	No additional findings
Korpilahti et al. (2007)	/Anna/ in neutral way for 740 ms	/Anna/ in commanding way for 740 ms /Anna/ in angry way for 740 ms	Emotional prosody Emotional prosody	Early‐MMN amplitude in AS children −	− −	Later‐MMN amplitude in AS children −	Later‐MMN in right hemisphere (*p* = 0.041) in AS children −	− −	Early‐MMN latency in AS children −	No additional findings No MMN differences of fathers of AS children compared to those of TD peers; early‐MMN in fathers of AS children in the right hemisphere (*p* = 0.001); later‐MMN in fathers of AS children in right hemisphere
Kujala et al. (2010)	Finnish syllables /te:/ or /pi:/ for 170 ms	Change by ±8% Vowel was shortened to 100 ms Changed by ±6 dB SPL /ti:/ for 170 ms /pe:/ for 170 ms	Syllable frequency Duration Intensity Consonant change Vowel change	− − Centro‐parietal region (*p* < 0.05) − −	Frontal region (*p* < 0.05) − − − −	− − − − +	− − − − −	− − − − −	− + − − +	No statistically significant MMN responses were elicited in either groups
Lassen et al. (2022)	1000 Hz tone for 50 ms	1200 Hz (50 ms), 1000 Hz (100 ms), 1200 Hz (100 ms)	Sound frequency, duration, and combined frequency‐duration	−	*p* = 0.026 (main effect of group, reduced MMN amplitude)	−	−	−	−	MMN amplitude significantly associated with adaptive functioning (ABAS‐GAC) in ASD (*p* = 0.0003). MMN response captured at Fz, FCz, Cz; P3a not different between groups.
Lepistö et al. (2005)	Vowels /a/ and /o/ and their non‐speech counterparts	Standard stimuli for 190 ms in a low pitch (113 Hz) Standard stimuli for 104 ms in low pitch (113 Hz) Standard stimuli for 190 ms in high pitch (125 Hz) Standard stimuli for 104 ms in high pitch	Duration and sound frequency Duration and sound frequency Duration and sound frequency Duration and sound frequency	Parietal MMN amplitudes in sound frequency changes in speech (*p* < 0.01) and non‐speech (*p* < 0.02) conditions − − −	Non‐speech duration deviants (*p* < 0.05) − − −	Duration or vowel changes in speech condition and vowel changes in non‐speech condition − − −	Non‐speech vowel deviants (*p* < 0.03) − − −	− − − −	− − − −	No additional findings
Lepistö et al. (2006)	Vowels /a/ and /o/ and their non‐speech counterparts	Standard stimuli for 190 ms in a low pitch (113 Hz) Standard stimuli for 104 ms in low pitch (113 Hz) Standard stimuli for 190 ms in high pitch (125 Hz) Standard stimuli for 104 ms in high pitch	Duration and sound frequency Duration and sound frequency Duration and sound frequency Duration and sound frequency	Right hemisphere (*p* < 0.06); parietal region of right hemisphere (*p* < 0.02) for sound‐frequency changes in speech condition − − −	Left hemisphere (*p* < 0.02); speech duration deviants (*p* < 0.01); non speech duration deviants (*p* < 0.02) − − −	− − − −	− − − −	Vowel deviants (*p* < 0.03) in both conditions − − −	− − − −	No additional findings
Lepistö et al. (2008)	Finnish vowels /a/, /e/, /i/, /o/, /u/	Standard stimuli presented at 100, 112, 123, 135, 149 or 166 Hz Finnish vowels /a/, /e/, /i/, /o/, /u/ Same vowel presented at a different pitch amongst 100, 112, 123, 135, 149 or 166 Hz Different vowel, same pitch	Sound frequency Phoneme identity Sound frequency Phoneme identity	Pitch changes in constant feature and varying feature conditions (*p* < 0.009); phoneme changes in the constant feature condition (*p* < 0.003); centro‐parietal amplitudes for pitch and phoneme changes (*p* < 0.025)	−	Varying feature condition (*p* = 0.91); phoneme changes in varying feature condition (*p* = 0.045)	−	−	+	No additional findings
Lepistö et al. (2009)	Pure tone 50 ms at 52 dB	Odd ball condition: pure tone for 50 ms at 67 dB Segregated condition: Intervening tones were 2637 Hz tones of four intensity values (47, 57, 62, and 72 dB). *p* < 0.02 Integrated condition: 4 intervening tones at 523 Hz	Intensity Intensity Intensity	− − −	− *p* < 0.02 −	Oddball condition − −	− − −	− − −	+ − −	MMN responses by both groups in all conditions except Integrated condition. No between group differences in MMN scalp distribution.
Lindström et al. (2016)	Finish word /Saara/ in neutral way for 577 ms	/Saara/ in commanding way for 538 ms /Saara/ in sad way for 775 ms /Saara/ in scornful way for 828 ms	Emotional prosody and duration Emotional prosody and duration Emotional prosody and duration	− − −	− − Frontal (*p* < 0.01) and central (*p* < 0.05) areas	− − −	− − −	− − −	+ − −	No additional findings
Lindström et al. (2018)	Finish word /Saara/ in neutral way for 577 ms	/Saara/ in commanding way for 538 ms /Saara/ in sad way for 775 ms /Saara/ in scornful way for 828 ms	Emotional prosody and duration Emotional prosody and duration Emotional prosody and duration ration	− − −	Centro‐parietal region (*p* = 0.045) for scornful deviants − −	− − −	− − −	− − −	− − −	No additional findings
Lortie et al. (2017)	Finger clicking (1981 Hz) or mouth sucking sounds (5857 Hz) for 150 ms	Acoustically matched control stimuli replicating the natural sounds; same sounds with different peak frequency (3876 Hz), peak latency (19 ms)	Duration and intensity	−	Biological sounds (*p* < 0.05)	−	−	−	−	No additional findings
Matsuba et al. (2024)	Syllable /da/	Syllable /ba/	Phoneme identity	−	−	No between‐group difference in MMN amplitude	Earlier MMN latency in ASD (*p* = 0.004)	−	−	Larger P1 amplitudes in ASD (*p* = 0.002); MMN amplitude correlated with Attention to Detail (r = −0.596, *p* = 0.001) and Sensory Over responsivity (r = −0.479, *p* = 0.012).
Matsuzaki et al. (2017)	300 ms sinusoidal tone: 300 Hz	300 ms sinusoidal tone: 700 Hz	Sound frequency	−	−	−	−	In both temporal hemispheres and frontal areas, ASD with divergent auditory sensitivity had longer MMF latencies than the other two groups (TD peers and ASD without different auditory sensitivity) (*p* < 0.05).	−	In both temporal hemispheres and frontal areas, ASD with divergent auditory sensitivity had longer M100 latencies than the other two groups (TD peers and ASD without different auditory sensitivity) (*p* < 0.05).
Oram Cardy et al. (2005)	300 Hz tone for 300 ms /a/ at 100 Hz for 300 ms	700 Hz tone//u/ /u/ at 100 Hz for 300 ms	Sound frequency Phoneme identity	− −	− −	+ −	− −	*p* = 0.015 −	− −	No additional findings
Roberts et al. (2011)	300 Hz tone//a/	700 Hz for 300 ms	Sound frequency	−	−	−	−	*p* < 0.001	−	ASD + LI showed prolonged MMF latency (by ~50 ms) compared to ASD‐LI (*p* < 0.01).
Ruiz‐Martinez et al. (2020)	/a/ for 300 ms	/u/ for 300 ms	Phoneme identity	−	*p* < 0.05	−	−	−	−	
Sanglakh Ghoochan Atigh et al. (2024)	1000 Hz tone (standard duration: 100 ms; standard ISI: 1000 ms)	Duration deviant: 50 ms; ISI deviant: 500 ms	Sound duration and inter‐stimulus interval (ISI)	−	Diminished MMN amplitude at Cz (*p* = 0.003) and marginally at Fz (*p* = 0.050)	−	−	Prolonged MMN latency in ASD at Fz (*p* = 0.002) and Cz (*p* = 0.003)		No effect of deviant type (duration vs. ISI) on MMN; no group × deviant interaction.
Sawada et al. (2008)	Tone burst at 1000 Hz	Tone bursts at 1100 Hz	Sound frequency	−	−	−	−	−	−	Score on PDD + ADHD‐like symptoms measure correlated with MMN amplitudes at Cz (*r* = −0.81, *p* < 0.01) and Pz (*r* = −0.80, *p* < 0.01) and MMN latency at Fz (*r* = 0.57, *p* < 0.1) and Cz (*r* = 0.56, *p* < 0.1).
Schall et al. (2024)	1 kHz tone//da/	1.2 kHz tone//ga/	Sound frequency, duration, and phoneme identity	−	Significantly smaller phoneme MMN in ASD (7.9 and 10.4 years cohorts)	−	Shorter MMN latency for duration deviants in ASD (7.9 years cohort)	−	−	MMN amplitude increased with age in TD; phonetic MMN significantly correlated with age in TD (r = −0.66, *p* < 0.01); no group differences in MMN latency for frequency or phoneme deviants.
Vlaskamp et al. (2017)	Tone at 1000 Hz, intensity of 75 dB SPL and was presented for 50 ms	1200 Hz tone for 50 ms 1000 Hz for 100 ms 1200 Hz tone for 100 ms	Sound frequency Duration Sound frequency and duration	−	Duration (*p* = 0.004) and Frequency duration (*p* = 0.009) deviants	−	−	−	+	No additional findings
Weismüller et al. (2015)	1 kHz‐tones at 80 dB SPL for 50 ms Phoneme /ba/ with 20 ms VOT	1.2 kHz tones at 80 dB SpL for 50 ms 1 kHz tones at 80 dB SpL for 100 ms 1 kHz tones at 90 dB SpL for 100 ms Phoneme /pa/ with 60 ms VOT	Sound frequency Duration Intensity Phoneme identity	− − − −	− − − −	Fz electrode − − −	− − − −	− − − −	− − − −	In ASD, larger MMN amplitudes at Fz correlated with age for duration (*r* = −0.53, *p* = 0.022) and intensity deviants (*r* = −0.51, *p* = 0.031). For frequency deviants, older age correlated with left hemispheric lateralization in TD peers (*r* = 0.52; *p* = 0.47), and right hemispheric lateralization in ASD (*r* = −0.50; *p* = 0.35)
Yu et al. (2015)	Pure tone of 216 Hz for 350 ms Lexical real word condition: /bai2/ with rising tone for 350 ms Lexical non‐word condition: /rai/ with a rising tone for 350 ms Hummed version of /bai2/ with a rising tone for 350 ms	Pure tone of 299 Hz for 30 ms Lexical real word condition: /bai4/ with a falling tone for 350 ms Lexical non‐word condition: /rai/ with a falling tone for 350 ms Hummed version of /bai4/ with a falling tone for 350 ms	Sound frequency Sound frequency Sound frequency Sound frequency	Cz electrode (*p* = 0.033) − − Cz electrode (*p* = 0.013)	− Fz electrode (*p* = 0.032) − −	− − + −	Cz electrode (*p* = 0.05) − Fz electrode (*p* = 0.042) −	− − − −	− + − +	No additional findings
Zhang et al. (2018)	The word MOther, in which /MO/ syllable was stressed	The word moTHER, in which /THER/ syllable was stressed	Lexical stress	Right hemisphere (*p* < 0.05)	Left hemisphere (*p* < 0.05)	−	−	−	−	No additional findings
Zhang et al. (2019)	Cantonese syllable /ga1/ at 282.9 Hz for 170 ms Pure tone at 1000 Hz, 75 dB for 100 ms	Cantonese syllable /ga2/ at 236 Hz and /ga3/ at 221.7 Hz, each for 170 ms Pure tone at 1200 Hz, 75 dB for 100 ms	/ga2/ pitch contour, /ga3/ pitch Height Sound frequency	− −	Pitch contour and (*p* = 0.037) and pitch height (*p* = 0.017) deviants at Fz electrode −	− Fz electrode	− −	− −	− −	No additional findings

*Note:* “−”, not applicable; “+”, present.

Abbreviations: ADHD, attention deficit hyperactivity disorder; AS, Asperger's syndrome; ASD, autism spectrum disorder; ASD + LI, autism spectrum disorder with language impairment; ASD‐LI, autism spectrum disorder without language impairment; Cz, central midline electrode; dB SPL, decibel sound pressure level; Fz, frontal midline electrode; Hz, hertz; ISI, inter‐stimulus interval; LE, left ear; LI, language impairment; M1/M2, left/right mastoid electrodes; MMF, mismatch field; MMN, mismatch negativity; ms, millisecond; RE, right ear; TD, typically developing; VOT, voice onset time.

**TABLE 5:** Auditory MMN and MMF responses in adolescents.

Stimuli characteristics				Study findings						
Study	Standard stimulus	Deviant stimulus	Difference between stimuli	Enhanced MMN amplitude or MMF power in ASD cases compared to TD peers	Attenuated MMN amplitude or MMF power in ASD cases compared to TD peers	No between‐group difference in MMN amplitude or MMF power	Earlier MMN or MMF latency in ASD cases compared to TD peers	Prolonged MMN or MMF latency in ASD cases compared to TD peers	No between‐group difference in MMN and MMF latencies	Other findings
Da Silva Mayerle et al. (2023)	1000 Hz tone burst	2000 Hz tone burst	Sound frequency	Increased MMN amplitude in adolescents with ASD (*p* = 0.015 RE, *p* = 0.002 LE)	−	−	−	Prolonged MMN latency in adolescents with ASD (*p* < 0.001 both ears)	−	Most adolescents with ASD reported auditory hypersensitivity; altered wave morphology observed in some adolescents.
Hudac et al. (2018)	1000 Hz tone or 750 Hz tone; counterbalanced between subjects	1000 Hz tone or 750 Hz tone	Sound frequency	−	−	+	−	−	−	Older children exhibited attenuated MMN compared to younger children (*p* = 0.0001)
Lassen et al. (2022)	1000 Hz tone, 75 dB, 50 ms	1200 Hz (freq), 100 ms (dur), 1200 Hz + 100 ms (freq+dur)	Sound frequency duration, and freq+dur deviance	−	Attenuated MMN amplitude across all deviant types (*p* = 0.026)	−	−	−	+	MMN amplitude associated with adaptive functioning (ABAS‐GAC) only in ASD group (*p* = 0.0003)
Ludlow et al. (2014)	Syllables /baj/ and /pai/ at 272 Hz for 330 ms	Word deviants: /bait/ and /paip/; pseudoword deviants: /baip/ and /pait/ for 485 ms	Syllable identity and duration	−	In frontal regions for words (*p* < 0.05) and pseudowords (*p* < 0.05); centro‐parietal regions for words (*p* < 0.05)	−	−	−	−	No additional findings
Mamashli et al. (2017)	50 ms complex tone consisting of 10 sinusoidal tones starting at 500 Hz with 25% frequency increments between them	50 ms complex Tone (combination of simple tones) at 500 Hz with 30% frequency increments between each tone	Sound frequency	−	−	+	−	−	+	Right hemispheric lateralization of MMF in ASD (*p* = 0.03)
Sokhadze et al. (2016)	1000 Hz sinusoidal tones for 100 ms	1300 Hz sinusoidal tones for 100 ms	Sound frequency	Baseline (*p* = 0.029)	Post auditory integration training intervention (*p* = 0.04)	Frontal MMN amplitude post intervention	−	−	−	No additional findings
Tecchio et al. (2003)	Tone bursts of 1000 Hz for 100 ms	Tone bursts of 1200 Hz for 100 ms	Sound frequency	−	*p* = 0.008	−	−	−	−	No additional findings

*Note:* “−”, not applicable; “+”, present.

Abbreviations: ABAS‐GAC, adaptive behavior assessment system (GAC scores); ASD, autism spectrum disorder; Hz, hertz; LE, left ear; MMF, mismatch field; MMN, mismatch negativity; ms, millisecond; RE, right ear; TD, typically developing.

**TABLE 6:** Auditory MMN and MMF responses in adults.

Stimuli characteristics				Study findings						
Study	Standard stimulus	Deviant stimulus	Difference between stimuli	Enhanced MMN amplitude or MMF power in ASD cases compared to TD peers	Attenuated MMN amplitude or MMF power in ASD cases compared to TD peers	No between‐group difference in MMN amplitude or MMF power	Earlier MMN or MMF latency in ASD cases compared to TD peers	Prolonged MMN or MMF latency in ASD cases compared to TD peers	No between‐group difference in MMN and MMF latencies	Other findings
Chien et al. (2018)	1000 Hz tone for 50 ms	1000 Hz tone for 100 ms; 1200 Hz tone	Duration deviant; frequency deviant	−	−	+	−	−	+	No additional findings
Fan and Cheng (2014)	Nonsense syllable /dada/ for 550 ms at 59 dB and control stimuli based on sinewaves using the nonvocal counterparts.	/dada/ in happy and angry tones, each for 550 ms at 59 dB and control stimuli based on sinewaves using the non‐vocal counterparts.	Emotional prosody	−	*p* < 0.05	−	−	−	+	Stronger MMN amplitudes for angry than happy deviants in TD peers (*p* < 0.001), but not in ASD (*p* = 0.67).
Goris et al. (2018)	Low frequency block: Five identical tones (xxxxx); high frequency block: Four identical and one non‐identical tones (xxxxy)	Low frequency block: Four identical and one non‐identical tones (xxxxy); high frequency block: Five identical tones (xxxxx)	Tone identity	−	Low frequency block (*p* = 0.02)	High‐frequency block	−	−	−	Larger MMN amplitude to low frequency blocks than high frequency blocks (*p* < 0.001) and this effect was larger in TD than ASD.
Haigh et al. (2023)	Tones: 1046.5, 1108.73, 1244.51 Hz; 50 ms duration	Change in pitch between tones	Pitch deviance (roving paradigm)	−	*p* < 0.001 (smaller MMN in SCZ than ASD & TD)	ASD vs. TD: not significant	−	−	−	Increased TTV in ASD and SCZ; MMN correlated with age; TTV negatively correlated with IQ in ASD.
Kasai et al. (2005)	Pure tone for 100 ms Japanese phoneme /a/ for 150 ms Japanese phoneme /a/	Pure tone for 50 ms Japanese phoneme /a/ for 100 ms Japanese phoneme /o/	Duration Duration Phoneme identity	− − −	− − −	+ − −	− − −	− − Left hemisphere (*p* < 0.05)	+ − −	MMF latency negatively correlated with neuroleptics and anticholinergic drugs in ASD.
Kujala et al. (2005)	Finish word /Saara/ in a neutral way for 577 ms	/Saara/ in a commanding way for 538 ms /Saara/ in a sad way for 775 ms /Saara/ in a scornful way for 828 ms	Emotional prosody and duration Emotional prosody and duration Emotional prosody and duration	− − −	Right‐hemispheric temporal–parietal electrodes (*p* < 0.03) − Right‐hemisphere (*p* < 0.04)	− + −	− − −	*p* < 0.01 − −	− + −	No additional findings
Kujala et al. (2007)	100 ms long harmonical tones composed of 500 Hz, 1000 Hz and 1500 Hz sinusoidal tones	470 Hz, 940 Hz, 1410 Hz or 530 Hz, 1060 Hz, 1590 Hz Increased/ decreased duration of the standard stimuli by 65 ms (+) or (−) 5 dB Right or left ear 10 ms gap between the 2 stimuli	Sound frequency Duration Intensity Location Gap	− Fz and Cz electrodes (*p* < 0.05) − − Fz and Cz electrode (*p* < 0.05)	− − − − −	+ − − − −	Oddball (*p* < 0.01) and multi‐feature (*p* < 05) conditions − − − −	− − − − −	− + + + +	No additional findings
Lepistö et al. (2007)	Vowels /a/, /o/	Vowels with the duration of 190 ms in a low pitch (113 Hz) Vowels with a duration of 104 ms in a low pitch (113 Hz) Vowels with a duration of 190 ms in a high pitch (125 Hz) Vowels with duration of 104 ms in a high pitch	Duration and sound frequency Duration and sound frequency Duration and sound frequency Duration and sound frequency	Right hemisphere (*p* < 0.001), left (*p* < 0.04), midline (*p* < 0.001) for speech‐pitch deviants; frontal MMN for speech‐duration deviants (*p* < 0.05) − − −	− − − −	− − − −	− − − −	− − − −	− − − −	No additional findings
Matsuzaki et al. (2019)	Vowel /a/ for 300 ms	Vowel /u/ for 300 ms	Phoneme identity	−	−	+	−	*p* < 0.05	−	Right‐hemispheric lateralization of MMF power was reported in autistic cases
Randeniya et al. (2022)	500 Hz tone in narrow and broad contexts	2000 Hz tone	Frequency deviance in stochastic oddball	−	−	+	−	−	+	MMN modulation by sensory sensitivity (SPQ) not by AQ; delta‐MMN correlated with auditory sensitivity (SPQ); no correlation with IQ or ADOS; no group difference in delta‐MMN or delta‐N300.
Sato et al. (2025)	1000 Hz, 100 ms, 75 dB	1000 Hz, 50 ms, 75 dB	Duration	−	−	+	(Fz: *p* = 0.001; Cz: *p* = 0.004)	−	−	Shortened d‐MMN latency in ASD adults may reflect persistent auditory hypersensitivity; no correlation with AQ.

*Note:* “−”, not applicable; “+”, present.

Abbreviations: ADOS, autism diagnostic observation schedule; AQ, autism spectrum quotient; ASD, autism spectrum disorder; EQ, emotional quotient; IQ, intelligence quotient; MMF, mismatch field; MMN, mismatch negativity; SCZ, schizophrenia; SQP, sensory perception quotient; TD, typically developing; TTV, trial‐to‐trial variability.

We excluded studies that involved participants with syndromic forms of autism (e.g., Fragile X syndrome, Rett syndrome, tuberous sclerosis), used active oddball paradigms (to minimize attentional confounds), or recorded MMN or MMF responses in the presence of background noise.

Study selection was performed in two stages by two independent reviewers (S.C.‐S. and S.J.), with any discrepancies resolved through consultation with senior authors. In the first stage, titles and abstracts were screened against predefined eligibility criteria, leading to the exclusion of 513 records (Figure 1). In the second stage, full texts of the remaining 122 studies were reviewed, and 67 were excluded for not meeting the inclusion criteria. A total of 55 studies met all criteria and were included in the final review (Figure 1).

Data extraction was conducted independently by the same two reviewers. Any disagreements were resolved by senior authors. Extracted data included: (1) participant demographics (age, sex/gender, sample size, diagnosis), (2) physical properties of the standard and deviant stimuli, and (3) MMN and MMF amplitude and power, respectively, and latency values.

### 2.3 Quality Assessment

The methodological quality of the included studies was independently assessed by two reviewers (S.C.‐S. and S.J.) using the Newcastle–Ottawa Scale (NOS) (Wells et al., n.d., http://www.ohri.ca/programs/clinical_epidemiology/oxford.htm). The NOS evaluates studies across three domains: (1) selection of the study groups; (2) comparability between groups; and (3) assessment of outcomes. Higher scores within each domain, as well as the total score, reflect greater methodological quality. Any disagreements between reviewers were resolved through discussion. If consensus could not be reached, senior authors were consulted to arbitrate and finalize the assessment.

## 3 Results

We conducted a narrative synthesis of the included studies (Figure 1) in accordance with the SWiM reporting guidelines (Campbell et al. 2020), due to substantial methodological heterogeneity across studies. The SWiM approach, developed to complement the PRISMA framework (Page et al. 2021), provides a structured method for synthesizing findings in the absence of meta‐analytic pooling.

### 3.1 Demographics and Characteristics of Included Studies

The included studies examined auditory MMN and MMF responses more extensively in children (*n* = 39) than in adolescents (*n* = 7) or adults (*n* = 11) with autism (Tables 1, 2, 3), yielding a total of 55 unique studies across developmental stages. Thus, the evidence base was heavily weighted toward childhood cohorts, with comparatively fewer studies addressing adolescence and adulthood, limiting the ability to draw continuous developmental inferences across age ranges.

A substantial proportion of study samples over‐represented males, consistent with the higher prevalence of autism in males but also reflecting sampling imbalance (Tables 1, 2, 3). Eight studies included exclusively male participants (Ferri et al. 2003; Lindström et al. 2018; Ludlow et al. 2014; Mamashli et al. 2017; Matsuzaki et al. 2017, 2019; Sawada et al. 2008; Weismüller et al. 2015) (Tables 1, 2, 3). This sex imbalance likely limits the generalizability of findings to the broader autistic population, particularly with respect to potential sex‐related differences in auditory mismatch processing.

Across MMN studies, EEG recordings were obtained using variants of the International 10–20 system, which positions electrodes at 10% or 20% intervals based on anatomical landmarks extending from the nasion to the inion and along the lateral coronal plane (Acharya et al. 2016; Klem et al. 1999). Electrode montages varied across studies, with some focusing primarily on midline frontal (Fz), frontocentral (FCz), or central (Cz) sites, whereas others sampled bilateral frontal (F), central (C), parietal (P), or temporal (T) electrodes. In contrast, MMF studies employed a range of whole‐head MEG systems with differing sensor configurations and source reconstruction approaches, introducing additional methodological heterogeneity in terms of spatial resolution and regional specificity. As a result, the scalp or cortical regions used to extract MMN or MMF responses differed substantially across studies.

In terms of stimulus design, studies using non‐speech stimuli typically presented deviant tones that differed from standard tones along one or more acoustic dimensions, including frequency, duration, intensity, inter‐stimulus interval, or combinations of these features. By contrast, studies employing speech stimuli introduced deviants that reflected either low‐level acoustic changes (e.g., vowel or syllable duration, pitch, or intensity) or higher‐order phonetic and linguistic contrasts (e.g., vowel or consonant identity, lexical stress, or emotional prosody). This variability in stimulus type, acoustic complexity, and deviance structure likely contributed to inconsistencies observed across empirical findings in MMN and MMF amplitude, latency, and spatial distribution.

To preserve passive listening conditions during oddball paradigm presentations, most studies instructed participants to engage in an unrelated background activity. The most common approach was watching a silent movie or muted cartoon, which was reported in the majority of EEG and MEG studies across age groups (Tables 1, 2, 3). Other strategies included imagining or reading a pictured book (e.g., Abdeltawwab and Baz 2015; Sawada et al. 2008), fixating on a visual point or cross (e.g., Haigh et al. 2023; Kemner et al. 1995; Matsuzaki et al. 2017), or viewing written instructions prompting participants to remain still during stimulus presentation (e.g., Sokhadze et al. 2016; Vlaskamp et al. 2017). Two studies (Berman et al. 2016; Tecchio et al. 2003), did not report the nature of the background activity used to maintain passive engagement.

### 3.2 Auditory MMN and MMF in Autistic Children Using Non‐Speech Stimuli

#### 3.2.1 Mismatch Response Amplitude

Across child non‐speech paradigms, many studies reported smaller MMN amplitudes in autistic children than in TD peers when standards and deviants differed in basic acoustic features of repetitive tones or tone bursts (Tables 1 and 4). Reductions in MMN amplitude were most consistently reported for frequency contrasts. Several EEG studies used 1000 Hz standards with higher‐frequency deviants and observed attenuated MMN amplitudes in autistic children, including contrasts with 1500 Hz tone bursts (Abdeltawwab and Baz 2015), 1200 Hz tones (Di Lorenzo et al. 2020), and 2000 Hz tone bursts (Da Silva Mayerle et al. 2023). Gomot et al. (2011) similarly contrasted 1000 Hz standards with 1100 Hz deviants and reported reduced MMN amplitude at the right mastoid (M2) in autistic children. Lassen et al. (2022) extended these findings using 1000 Hz, 50 ms standards and deviants that differed in frequency (1200 Hz), duration (100 ms), or both, and reported reduced MMN amplitudes in autistic children across all tone‐based deviant types.

Reduced MMN amplitudes in autism were also reported for non‐speech duration contrasts, in which standards and deviants preserved frequency but differed in temporal length. Di Lorenzo et al. (2020) manipulated duration, presenting 1000 Hz standards (50 ms) and longer 1000 Hz deviants (100 ms), and reported smaller MMN amplitudes in autistic children compared to TD relatives. Vlaskamp et al. (2017) used 1000 Hz standards (75 dB SPL, 50 ms) and introduced deviants that either lengthened the tone (100 ms) or combined frequency and duration changes (1200 Hz, 100 ms). Both deviant types elicited reduced MMN amplitudes in autistic children compared to TD peers. Da Silva Mayerle et al. (2023) reported reduced MMN amplitude in autistic children for a frequency contrast (1000 Hz standards vs. 2000 Hz deviants). Sanglakh Ghoochan Atigh et al. (2024) used 1000 Hz standards (100 ms, ISI 1000 ms) and presented deviants that shortened duration (50 ms) or inter‐stimulus interval (500 ms), reporting smaller MMN amplitudes in autistic children at Cz, with marginal effects at Fz, relative to TD children. Huang et al. (2018) similarly reported attenuated MMN amplitudes for a duration deviant in which 295 Hz tones were presented for 250 ms as standards and extended to 350 ms as deviants, with responses quantified at the left mastoid (M1).

Stimulus context further modulated group differences in intensity‐based non‐speech paradigms. Lepistö et al. (2009) used 50 ms pure‐tone standards and manipulated both intensity and stream context. In the oddball condition, standards were presented at 52 dB and deviants at 67 dB, whereas in the segregated condition, intervening tones (2637 Hz) promoted stream separation and autistic children showed smaller MMN amplitudes than TD peers. In the integrated condition, intervening tones (523 Hz) promoted perceptual grouping, and neither autistic nor TD children exhibited a statistically significant MMN, indicating that the absence of a group difference reflected a lack of a measurable deviant–standard response in both groups rather than preserved processing in autism.

Reduced MMN amplitudes in autism were not restricted to pure‐tone paradigms. Lortie et al. (2017) used biologically relevant non‐speech sounds as standards—finger clicks (1981 Hz) or mouth‐suction sounds (5857 Hz)—and contrasted them with acoustically matched control stimuli that differed in spectral and temporal properties, including peak frequency and peak latency. That study reported smaller MMN amplitudes in autistic children than in TD peers, indicating that amplitude reductions extended to naturalistic non‐speech contrasts.

A smaller subset of studies reported larger MMN amplitudes in autistic children than in TD peers for specific non‐speech contrasts. Ferri et al. (2003) used 1000 Hz sinusoidal standards and 1300 Hz sinusoidal deviants and reported greater deviant–standard MMN amplitude at Cz in autistic children, while a separate novelty contrast using complex, non‐monotonal sounds did not yield a significant between‐group difference. Yu et al. (2015) similarly reported larger MMN amplitudes at Cz in autistic children for a pure‐tone frequency contrast using 216 Hz standards and 299 Hz deviants (350 ms duration).

Several studies reported no significant differences between autistic and TD participants in non‐speech MMN or MMF amplitude. Cary et al. (2024) used 1000 Hz standards and 1200 Hz deviants and reported comparable MMN amplitudes between groups. Čeponiene et al. (2003) used simple‐tone standards (458 Hz) and complex‐tone standards composed of multiple frequencies (458, 1370, 2054, and 3537 Hz), with deviants created by a 10% frequency increase, and reported no difference in amplitude between autistic and TD participants, with both groups exhibiting larger MMNs to complex than to simple tones. Kabil et al. (2023) reported no amplitude difference for tone bursts differing in frequency (1000 Hz vs. 2000 Hz). Oram Cardy et al. (2005) measured MMF responses to non‐speech tones (300 Hz standards vs. 700 Hz deviants) and reported no MMF amplitude difference for the tone condition. Zhang et al. (2019) included a pure‐tone frequency contrast (1000 Hz standards vs. 1200 Hz deviants) and reported no between‐group MMN amplitude difference at Fz.

Only a small number of studies explicitly examined age‐related patterns in non‐speech MMN amplitude within childhood cohorts. Abdeltawwab and Baz (2015) reported that, for a frequency contrast using 1000 Hz standards and 1500 Hz deviants, MMN amplitude increased with age within the autistic group relative to the TD group. Ferri et al. (2003) modeled MMN amplitude across age and reported electrode‐dependent developmental trajectories. At Fz and Cz sites, autistic children initially showed larger MMN amplitudes than TD peers, but these values approached TD levels at the upper end of the modeled age range. In contrast, between‐group differences persisted at Pz. Weismüller et al. (2015) examined non‐speech deviants that altered frequency (1.0 kHz vs. 1.2 kHz), duration (50 ms vs. 100 ms), or intensity (80 dB vs. 90 dB) and reported significant age–amplitude correlations within the autistic group for duration and intensity deviants, without identifying statistically significant difference in amplitude between autistic and TD participants. Sawada et al. (2008) presented tone bursts (1000 Hz standards vs. 1100 Hz deviants) in a PDD‐only cohort without a TD comparison group and therefore did not contribute autism–TD amplitude contrasts but reported that higher PDD and ADHD‐like symptom scores were associated with smaller MMN amplitudes at Cz and Pz.

#### 3.2.2 Mismatch Response Latency

Latency findings for non‐speech paradigms in children varied across studies, with reports of both earlier and prolonged MMN or MMF latencies in autistic children relative to TD peers, depending on the acoustic feature manipulated and on subgroup characteristics (Tables 1 and 4). Gomot et al. (2002) used a tone‐frequency contrast (1000 Hz standards vs. 1100 Hz deviants) and reported earlier MMN latencies in autistic children at Fz and both mastoids (M1/M2), indicating faster mismatch timing in that cohort. Schall et al. (2024) included a non‐speech tone condition and reported shorter MMN latency for duration deviants in autistic children within the younger subgroup (mean age 7.9 years), indicating that latency direction depended on age and deviant type within the same dataset.

In contrast, several studies reported prolonged MMN or MMF latencies in autistic children relative to TD peers for non‐speech contrasts. For example, Di Lorenzo et al. (2020) found longer MMN latencies in autistic children compared to TD participants for frequency deviants, using 1000 Hz, 50 ms standards and 1200 Hz, 50 ms deviants. Sanglakh Ghoochan Atigh et al. (2024) reported prolonged MMN latency at Fz and Cz for deviants that shortened stimulus duration (100 ms to 50 ms) and for deviants that shortened inter‐stimulus interval (1000 ms to 500 ms). Da Silva Mayerle et al. (2023) reported prolonged MMN latency for tone bursts differing in frequency (1000 Hz standards vs. 2000 Hz deviants). Kabil et al. (2023) similarly reported prolonged MMN latency for tone bursts using the same frequency contrast. MEG evidence also indicated prolonged timing in autism for non‐speech tones, as Oram Cardy et al. (2005) reported prolonged MMF latency for a tone‐frequency contrast (300 Hz standards vs. 700 Hz deviants). Huang et al. (2018) reported prolonged MMN latency for a tone‐duration contrast (295 Hz, 250 ms standards vs. 295 Hz, 350 ms deviants), quantified at the left mastoid (M1).

Several studies indicated that latency differences related more strongly to individual characteristics than to diagnosis alone. Jansson‐Verkasalo et al. (2003) used a non‐speech tone‐frequency contrast (1000 Hz standards vs. 1100 Hz deviants) and reported prolonged MMN latencies over both hemispheres in children with autism relative to TD peers. Matsuzaki et al. (2017) contrasted 300 Hz and 700 Hz sinusoidal tones and showed that autistic children with heightened auditory sensitivity exhibited longer MMF and M100 latencies than both TD children and autistic children without divergent sensitivity. Gomot et al. (2011), using 1000 Hz standards and 1100 Hz deviants, reported within‐autism variability such that autistic children with higher intolerance to change showed shorter MMN latencies at M1/M2 than autistic children with lower intolerance to change.

Other studies reported no significant between‐group differences in latency for non‐speech contrasts. Abdeltawwab and Baz (2015) reported comparable MMN latencies for 1000 Hz standards and 1500 Hz deviants. Cary et al. (2024) similarly reported no autism–TD latency difference for 1000 Hz versus 1200 Hz contrasts. Čeponiene et al. (2003) reported no latency differences for 10% frequency increments applied to both simple and complex tones. Vlaskamp et al. (2017) also reported no latency difference between autistic and TD participants despite reduced MMN amplitudes for duration and combined frequency–duration deviants. Lepistö et al. (2009) did not report consistent latency differences between autistic and TD participants across intensity and stream‐segregation contexts and noted that neither group exhibited a statistically significant MMN in the integrated condition.

#### 3.2.3 Mismatch Response Hemispheric Distribution

Across child non‐speech paradigms, most studies did not formally assess hemispheric lateralization using explicit left–right statistical contrasts because MMN was quantified at midline electrodes or derived using analytic approaches that did not permit direct hemispheric comparison (Tables 1 and 4).

Several EEG studies emphasized midline quantification, including recordings at Fz (Abdeltawwab and Baz 2015; Di Lorenzo et al. 2020), FCz (Cary et al. 2024; Lortie et al. 2017), and Cz or Pz (Ferri et al. 2003; Lassen et al. 2022; Sanglakh Ghoochan Atigh et al. 2024). Other studies referenced bilateral mastoids without conducting hemisphere‐specific statistical tests, including recordings at Fz with M1/M2 (Da Silva Mayerle et al. 2023; Gomot et al. 2002) or broader montages including mastoids (Gomot et al. 2011). As a result, although these studies reported group differences in MMN amplitude or latency, their designs constrained inference about hemispheric dominance. Lepistö et al. (2009), who employed a broad bilateral montage (F3/F4, FC3/FC4, C3/C4, CP3/CP4, P3/P4), explicitly reported no autism–TD differences in MMN scalp distribution across non‐speech intensity and stream‐segregation conditions.

In several cases, hemispheric interpretation was further limited by restricted reporting. Huang et al. (2018) quantified MMN only at the left mastoid (M1), precluding left–right comparison, and Kabil et al. (2023) did not specify electrode locations, preventing assessment of hemispheric distribution despite reporting latency effects.

A small subset of studies provided hemisphere‐relevant information but did not converge on a consistent autism–TD laterality pattern for non‐speech stimuli. Gomot et al. (2002), recording from Fz, M1, and M2, reported group differences in MMN generator distribution, with TD children showing a frontocentral maximum and autistic children showing relatively greater lateral involvement, without identifying a single dominant hemisphere. Gomot et al. (2011), using a broader montage (Fz, Cz, Pz, T3, T4, M1, M2), reported reduced MMN amplitude at the right mastoid (M2) in autistic children. Jansson‐Verkasalo et al. (2003), using an extensive bilateral montage (F3/F4, C3/C4, P3/P4, T3/T4, with midline Fz, Cz, Pz, Oz), reported prolonged MMN latencies over both hemispheres for a non‐speech tone‐frequency contrast (1000 Hz vs. 1100 Hz), alongside group differences in hemispheric weighting rather than a unidirectional lateralization effect.

MEG studies localized non‐speech MMF primarily to temporal cortical regions and generally did not report formal hemispheric contrasts. Oram Cardy et al. (2005) localized MMF responses to the left hemisphere for a tone‐frequency contrast (300 Hz standards vs. 700 Hz deviants), while Matsuzaki et al. (2017) reported MMF and M100 responses in bilateral temporal cortices and frontal regions, linking latency differences to individual auditory sensitivity profiles rather than hemispheric dominance.

Overall, across EEG and MEG child non‐speech paradigms, hemispheric findings were strongly shaped by electrode selection and analytic strategy. Studies that permitted lateralized interpretation described electrode‐ and cohort‐specific spatial patterns, rather than a single consistent hemispheric distribution distinguishing autistic from TD children.

### 3.3 Auditory MMN and MMF in Autistic Children Using Speech Stimuli

#### 3.3.1 Mismatch Response Amplitude

Across child speech paradigms, studies reported both reduced and enhanced MMN amplitudes in autistic children relative to TD peers, with effect direction depending on the speech unit and on the feature distinguishing standards from deviants (Tables 1 and 4). Investigated contrasts included vowel identity, consonant identity, voice‐onset time (VOT), lexical tone, lexical stress, emotional prosody, and pitch or fundamental frequency.

Several studies reported attenuated MMN amplitudes in autistic children for contrasts involving phoneme or vowel identity. Ruiz‐Martinez et al. (2020) observed smaller MMN amplitudes for a vowel‐identity contrast (/a/ standard vs. /u/ deviant). Schall et al. (2024) similarly reported reduced MMN amplitudes for a phoneme contrast using /da/ standards and /ga/ deviants. Emotional prosody paradigms also yielded reduced MMN amplitudes in autism across multiple datasets. Lindström et al. (2016) reported smaller MMN amplitudes for prosodic deviants embedded within the word /Saara/, in which commanding, sad, or scornful prosody deviated from a neutral standard. Lindström et al. (2018) further showed reduced MMN amplitude for the scornful‐prosody deviant relative to the neutral condition within the same lexical item. Using the word /Anna/, Korpilahti et al. (2007) reported early‐ and later‐MMN amplitude effects for emotional prosody contrasts (neutral standard vs. commanding/angry deviants), including a later‐MMN effect with right‐hemisphere specificity in the autism group.

In contrast, several speech paradigms did not reveal differences between autistic and TD participants in MMN amplitude. Kemner et al. (1995) reported comparable amplitudes for a diphthong contrast (/oy/ standard vs. /ay/ deviant). Matsuba et al. (2024) similarly found no between‐group difference for a consonant‐identity contrast (/da/ vs. /ba/). Weismüller et al. (2015) reported no significant amplitude difference for a VOT contrast using /ba/ (20 ms VOT) standards and /pa/ (60 ms VOT) deviants. Kujala et al. (2010) examined a broad set of Finnish syllable contrasts—including changes in pitch (±8%), duration (170 ms vs. 100 ms), intensity (±6 dB), consonant identity (/ti:/), and vowel identity (/pe:/)—and did not elicit statistically significant MMNs in either autistic or TD children, limiting between‐group interpretation. Charpentier et al. (2018) used neutral versus angry /a/ tokens, but the reported amplitude effects applied only to adults, and the child cohort did not contribute a speech‐related MMN amplitude difference.

A smaller subset of studies reported enhanced MMN amplitudes for specific speech contrasts, most consistently those involving pitch or fundamental frequency manipulations. Lepistö et al. (2005) and Lepistö et al. (2006) used Finnish vowels (/a/ and /o/) as standards and introduced deviants differing in pitch or duration. These studies reported larger MMN amplitudes in the autism group than in TD peers for certain speech pitch/frequency contrasts, whereas Lepistö et al. (2006) reported reduced MMN amplitudes for speech duration deviants. Lepistö et al. (2008) extended this approach by presenting Finnish vowels (/a, e, i, o, u/) across varying pitch contexts and reported larger MMN amplitudes in autistic children for some pitch‐based and phoneme‐identity contrasts, with directionality depending on whether the acoustic context was stable or variable across trials. Zhang et al. (2019) examined Cantonese lexical tone contrasts using /ga1/ standards and /ga2/ (pitch contour change) or /ga3/ (pitch height change) deviants and reported reduced MMN amplitude at Fz in autistic children. Zhang et al. (2018) investigated lexical stress using the contrast MOther (standard) versus moTHER (deviant) and identified a hemispherically differentiated pattern in autism, characterized by relatively stronger right‐hemisphere and weaker left‐hemisphere MMN amplitudes. Jansson‐Verkasalo et al. (2005) manipulated fundamental frequency within the Finnish word *anna* (280 Hz vs. 320 Hz) and reported reduced MMN amplitudes over frontal and central regions in children with autism.

Finally, several studies included both speech and non‐speech conditions, and synthesis of speech‐related MMN amplitude effects relies on contrasts explicitly described as speech. Čeponiene et al. (2003) included a Finnish vowel /o/ condition alongside tone paradigms and did not report a between‐group amplitude difference for the summarized frequency‐change contrasts. Green et al. (2020) tested both a vowel‐identity contrast (/ɑ/ vs. /u/) and a pure‐tone contrast, but they did not indicate a speech‐specific amplitude difference for the vowel condition. Huang et al. (2018) examined a Mandarin syllable /du/ (250 ms) with a vowel‐lengthening deviant (350 ms), but speech and non‐speech duration effects were combined in the reporting, preventing isolation of a speech‐specific MMN amplitude direction.

#### 3.3.2 Mismatch Response Latency

Latency findings in child speech paradigms were heterogeneous and depended on both the speech contrast and subgroup characteristics (Tables 1 and 4). Several studies reported earlier MMN latency in autistic children under specific conditions. Matsuba et al. (2024) reported shorter MMN latencies in autistic children than in TD peers for a consonant identity contrast using /da/ standards and /ba/ deviants. Green et al. (2020) similarly reported earlier MMN latency for a vowel identity contrast (/ɑ/ standards vs. /u/ deviants), but this effect was specific to autistic children with more pronounced language challenges, relative to autistic children without language difficulties and TD peers.

A smaller number of studies reported prolonged MMN latency in children with autism compared with TD peers. Jansson‐Verkasalo et al. (2005) reported longer MMN latencies for phoneme changes within the Finnish word /anna/ when the deviant differed from the standard in fundamental frequency (280 Hz vs. 320 Hz). Berman et al. (2016) similarly reported prolonged MMN latencies for phoneme changes in Finnish syllables (/taa/ standards vs. /kaa/ deviants). In that study, latency prolongation was observed alongside hemispheric differences in MMN distribution, but the latency effect itself was not restricted to a single hemisphere.

Several studies linked prolonged mismatch timing more strongly to language ability than to diagnostic group alone. Using MEG, Berman et al. (2016) measured MMF responses to vowel identity changes (/a/ standards vs. /u/ deviants), localized to the superior temporal gyrus, and reported longer MMF latencies in autistic children with more pronounced language challenges compared with autistic children without language difficulty. Oram Cardy et al. (2005) also reported prolonged MMF latency in autistic children relative to TD peers for a vowel contrast (/a/ vs. /u/ at 100 Hz), with activity localized to the left hemisphere. Roberts et al. (2011) examined a speech‐based frequency contrast in which the vowel /a/ differed in sound frequency (300 Hz standard vs. 700 Hz deviant) and reported prolonged MMF latency in autistic children, particularly in those with more pronounced language challenges.

Other speech studies reported no significant autism–TD differences in latency. Kemner et al. (1995) reported comparable MMN latencies for a diphthong contrast (/oy/ standards vs. /ay/ deviants). Weismüller et al. (2015) likewise reported no latency difference for a voice‐onset‐time contrast (/ba/ with 20 ms VOT vs. /pa/ with 60 ms VOT). Lindström et al. (2016) reported no group difference in latency for emotional prosody contrasts within the word /Saara/, and Schall et al. (2024) reported no autism–TD latency difference for a phoneme contrast (/da/ standards vs. /ga/ deviants), despite observing amplitude differences.

Finally, some studies emphasized condition‐ and electrode‐specific timing effects rather than a single group‐level latency shift. Yu et al. (2015) examined Mandarin lexical tone contrasts in which standards and deviants differed in tone direction within real‐word (/bai2/ vs. /bai4/), non‐word (/rai/ rising vs. falling), and hummed syllable contexts, and reported latency effects that varied across conditions and recording sites. Huang et al. (2018) reported prolonged MMN latency in analyses that combined speech vowel‐lengthening (/du/) and non‐speech tone‐duration contrasts, but they did not separate latency outcomes by stimulus class, precluding speech‐specific attribution. Charpentier et al. (2018) did not report a child‐specific speech latency effect for neutral versus angry /a/ tokens.

#### 3.3.3 Mismatch Response Hemispheric Distribution

Across child speech paradigms, many studies did not formally test hemispheric lateralization because they quantified MMN/MMF at midline electrodes or used analytic strategies that did not implement explicit left–right statistical contrasts (Tables 1 and 4). Several EEG studies emphasized midline electrodes. For example, Fz in Schall et al. (2024) and Zhang et al. (2019) (montage: Fz/F3/F4, Cz/C3/C4, Pz/P3/P4, M1/M2), whereas Lindström et al. (2018), centered their analyses on Pz. Other studies reported broader regional effects (frontal, central, centro‐parietal) without conducting hemisphere‐specific comparisons (Lindström et al. 2016, 2018). These approaches supported autism–TD comparisons of MMN amplitude or latency but constrained inference about hemispheric dominance because they either collapsed activity across hemispheres or did not test homologous left–right pairs statistically.

When studies explicitly assessed hemispheric effects, laterality depended on the speech unit and on the feature distinguishing the standard and deviant. Jansson‐Verkasalo et al. (2003) used a bilateral montage (F3/F4, C3/C4, P3/P4, T3/T4, with midline Fz, Cz, Pz, Oz) and examined phoneme changes in Finnish syllables (/taa/ standards vs. /kaa/ deviants). The study reported right‐hemisphere lateralization in children with autism, with larger right‐ than left‐hemisphere MMN, while TD peers showed the opposite hemispheric weighting pattern. Lepistö et al. (2006) also used a broad bilateral montage (F3/C3/T3/TP3, F4/C4/T4/TP4, with midline Fz/Cz/Pz) and reported a right‐hemisphere‐dominant MMN distribution in children with autism for speech pitch/fundamental‐frequency changes within vowels (/a/, /o/). In the same study, speech duration deviants elicited reduced MMN amplitudes over the left hemisphere and midline in the autism group, indicating feature‐specific lateralization rather than a unitary hemispheric shift.

Other speech features also showed right‐weighted patterns when analyses permitted hemispheric interpretation. Korpilahti et al. (2007) recorded with a bilateral montage (F3/F4, C3/C4, CP5/CP6, P3/P4, with midline Fz/Cz/Pz) and identified a later‐MMN amplitude effect in the right hemisphere for emotional prosody contrasts within /Anna/ (neutral standard vs. commanding/angry deviants). Zhang et al. (2018) recorded over bilateral temporoparietal sites (CP3/CP4, T7/T8, P7/P8, TP9/TP10) and reported right‐hemisphere enhancement accompanied by left‐hemisphere attenuation of MMN amplitude for lexical stress contrasts (MOther vs. moTHER), consistent with a hemispheric redistribution rather than a uniform global change.

MEG studies in child speech paradigms primarily emphasized source localization rather than hemisphere‐level statistical contrasts. Berman et al. (2016) localized MMF responses to the superior temporal gyrus for vowel identity changes (/a/ vs. /u/), Roberts et al. (2011) localized MMF to the superior temporal gyrus for a speech‐based frequency contrast using /a/ as both standard and deviant (300 Hz vs. 700 Hz), and Oram Cardy et al. (2005) localized mismatch activity to the left hemisphere for a vowel contrast (/a/ vs. /u/ at 100 Hz). These studies did not report formal left–right statistical contrasts for speech conditions.

Taken together, the child speech literature indicated that studies that explicitly assessed laterality most often reported right‐hemisphere weighting in autistic children, particularly for contrasts involving phoneme identity (Jansson‐Verkasalo et al. 2003), pitch/fundamental frequency (Lepistö et al. 2006), emotional prosody (Korpilahti et al. 2007), and lexical stress (Zhang et al. 2018). Evidence for left‐hemisphere involvement appeared more narrowly and in a feature‐specific manner, most clearly for speech duration deviants (Lepistö et al. 2006) or in TD comparison patterns (Jansson‐Verkasalo et al. 2003). Overall, hemispheric effects in child speech paradigms reflected stimulus‐ and analysis‐dependent spatial organization rather than a single uniform lateralization pattern.

### 3.4 Auditory MMN and MMF in Autistic Adolescents Using Non‐Speech and Speech Stimuli

#### 3.4.1 Mismatch Response Amplitude

Across adolescent paradigms, studies reported attenuated, enhanced, and unchanged MMN/MMF amplitudes in autism relative to TD peers, and the direction of effects depended on stimulus class and on the feature that differed between standards and deviants (Tables 2 and 5). Several non‐speech studies reported reduced MMN/MMF amplitude for tone‐based contrasts. Lassen et al. (2022) presented 1000 Hz, 75 dB, 50 ms standards and used deviants that changed frequency (1200 Hz), duration (100 ms), or frequency and duration jointly (1200 Hz, 100 ms), reporting smaller MMN amplitudes in autistic adolescents across deviant types at Fz, FCz, and Cz. Tecchio et al. (2003) contrasted tone bursts that differed in frequency (1000 Hz, 100 ms standards vs. 1200 Hz, 100 ms deviants) and reported reduced MMF amplitude in autistic adolescents, localizing responses to the midtemporal lobe contralateral to the stimulated ear.

Other adolescent non‐speech paradigms did not detect autism–TD amplitude differences. Hudac et al. (2018) used a two‐tone frequency oddball in which 1000 Hz and 750 Hz tones were counterbalanced as standards and deviants across participants and reported no MMN amplitude difference between autistic and TD participants at central medial electrodes, while also showing that older participants exhibited smaller MMN amplitudes than younger participants within the sample. Mamashli et al. (2017) presented 50 ms complex tones composed of ten sinusoids starting at 500 Hz and manipulated spectral structure by using 25% frequency increments for standards and 30% increments for deviants; the study reported no autism–TD difference in MMF power with sources localized to the superior temporal gyrus, middle temporal gyrus, and inferior frontal gyrus.

A smaller subset of non‐speech studies reported enhanced MMN amplitude in autism for frequency contrasts. Da Silva Mayerle et al. (2023) contrasted tone bursts that differed in frequency (1000 Hz standards vs. 2000 Hz deviants) and reported increased MMN amplitude in autistic adolescents for both ears, quantified at Fz with bilateral mastoids (M1/M2). Sokhadze et al. (2016) contrasted 1000 Hz and 1300 Hz sinusoidal tones (100 ms) and reported larger MMN amplitudes in autistic adolescents than TD peers at baseline at Fz, FC1, FC2, and Cz. Following auditory integration training, the same study reported an amplitude change relative to baseline and described post‐training frontal MMN amplitude effects, but there is no persistent autism–TD amplitude difference after training.

Speech paradigms in adolescents were represented primarily by Ludlow et al. (2014). That study presented syllable standards (/baj/ and /pai/; 272 Hz, 330 ms) and introduced deviants that changed both identity and duration by presenting word deviants (/bait/ and /paip/; 485 ms) and pseudoword deviants (/baip/ and /pait/; 485 ms). The study reported smaller MMN amplitudes in autistic adolescents than TD peers over frontal regions for both word and pseudoword deviants and over centro‐parietal regions for word deviants, with MMN quantified at CPz and regional effects described in the report.

#### 3.4.2 Mismatch Response Latency

Latency outcomes in adolescent studies were reported for a limited subset of paradigms and were driven primarily by non‐speech contrasts, yielding a mixture of null findings and evidence for prolonged mismatch timing in autism (Tables 2 and 5). Lassen et al. (2022) examined MMN latency across frequency, duration, and combined frequency–duration deviants (1000 Hz standards vs. 1200 Hz and/or 100 ms deviants), recorded at Fz, FCz, and Cz, and reported no significant autism–TD differences in MMN latency across any deviant type. Mamashli et al. (2017) similarly reported no autism–TD difference in MMF latency for a complex‐tone frequency‐increment contrast, with responses localized to the superior temporal gyrus, middle temporal gyrus, and inferior frontal gyrus.

In contrast, Da Silva Mayerle et al. (2023) reported prolonged MMN latency in autistic adolescents relative to TD peers for a tone‐burst frequency contrast (1000 Hz standards vs. 2000 Hz deviants). Latency effects were observed for both ears, with recordings at Fz and bilateral mastoids (M1/M2), indicating delayed mismatch timing in autism for this specific non‐speech paradigm.

Other adolescent studies did not report autism–TD latency outcomes for their speech or non‐speech contrasts (Tables 2 and 5). These included studies emphasizing amplitude measures or intervention effects (Hudac et al. 2018; Sokhadze et al. 2016), studies focusing on lexical or syllabic processing without latency analyses (Ludlow et al. 2014), and MEG studies that localized mismatch responses without reporting latency comparisons (Tecchio et al. 2003). Consequently, conclusions regarding latency differences in adolescence were constrained by limited reporting and were informed primarily by a small number of non‐speech paradigms.

#### 3.4.3 Mismatch Response Hemispheric Distribution

Across adolescent speech and non‐speech paradigms, most investigations did not provide formal hemispheric comparisons because they quantified MMN at midline electrodes or localized MMF to regions without reporting explicit left–right statistical tests (Tables 2 and 5). EEG studies relied on midline or near‐midline recordings, including central medial electrodes (Hudac et al. 2018), Fz/FCz/Cz (Lassen et al. 2022), CPz (Ludlow et al. 2014; speech syllable/word and pseudoword contrasts), Fz with mastoids M1/M2 (Da Silva Mayerle et al. 2023), and Fz/FC1/FC2/Cz (Sokhadze et al. 2016). These electrode configurations supported autism–TD comparisons of MMN amplitude and, in some cases, latency, but they constrained hemispheric inference because they did not include bilateral homologous scalp measures used for direct left–right statistical contrasts.

MEG studies provided the clearest hemisphere‐relevant information in adolescence. Mamashli et al. (2017) used a whole‐head VectorView MEG system and localized MMF responses to the superior temporal gyrus, middle temporal gyrus, and inferior frontal gyrus, reporting right‐hemisphere lateralization of MMF responses in autistic adolescents for the complex‐tone frequency‐increment contrast. Tecchio et al. (2003) used a 28‐channel MEG system and localized MMF responses to the midtemporal lobe contralateral to the stimulated ear, describing contralateral auditory organization rather than an autism‐specific hemispheric asymmetry.

Overall, adolescent hemispheric evidence depended strongly on method. EEG studies using midline‐focused electrode sets did not permit robust lateralization assessment, whereas MEG studies that evaluated hemispheric distribution reported right‐lateralized MMF effects in a specific non‐speech complex‐tone paradigm, with no comparable hemisphere‐resolved evidence reported for the adolescent speech paradigms (Tables 2 and 5).

### 3.5 Auditory MMN and MMF in Autistic Adults Using Non‐Speech Stimuli

#### 3.5.1 Mismatch Response Amplitude

Across adult non‐speech paradigms, most studies reported no consistent autism–TD difference in MMN or MMF amplitude when standards and deviants differed in tone frequency, duration, or pitch‐related features (Tables 3 and 6). Chien et al. (2018) contrasted 1000 Hz standards with deviants that differed either in duration (50 ms vs. 100 ms) or frequency (1000 Hz vs. 1200 Hz) and reported comparable MMN amplitudes between autistic and TD adults across both deviant types. Randeniya et al. (2022) similarly reported no between‐group MMN amplitude difference in a stochastic oddball paradigm using 500 Hz standards (presented in narrow and broad contexts) and 2000 Hz deviants. Sato et al. (2025) examined a tone‐duration contrast (1000 Hz, 100 ms standards vs. 1000 Hz, 50 ms deviants) and likewise found no autism–TD amplitude difference. Haigh et al. (2023), using a roving pitch‐deviance design in which pitch varied across successive tones, also reported no group difference in MMN amplitude.

Several studies, however, identified condition‐dependent amplitude reductions in autism rather than a uniform group effect. Goris et al. (2018) manipulated tone‐identity structure across blocks and reported reduced MMN amplitude in autism specifically in the low‐frequency block condition, whereas the high‐frequency block did not show an autism–TD difference. Kujala et al. (2007) employed a multi‐feature harmonic‐tone paradigm and reported enhanced MMN amplitudes in autism at frontocentral sites (Fz and Cz) for specific non‐speech deviants, including sound frequency and duration, while other deviant dimensions did not yield consistent group separation.

MEG evidence converged with the EEG findings in showing limited amplitude effects. Kasai et al. (2005) examined a pure‐tone duration contrast (100 ms standard vs. 50 ms deviant) and reported no autism–TD difference in MMF amplitude.

#### 3.5.2 Mismatch Response Latency

Latency findings in adult non‐speech paradigms were heterogeneous but most often indicated no autism–TD difference, with a small number of studies reporting directionally specific timing effects (Tables 3 and 6). Chien et al. (2018) reported no autism–TD difference in MMN latency for either a tone‐duration deviant (1000 Hz, 50 ms vs. 100 ms) or a tone‐frequency deviant (1000 Hz vs. 1200 Hz). Randeniya et al. (2022) similarly found no autism–TD difference in MMN latency for a stochastic frequency contrast using 500 Hz standards (narrow and broad contexts) and 2000 Hz deviants. Kujala et al. (2007) also reported no between‐group latency differences across the non‐speech deviant types in their multi‐feature harmonic‐tone paradigm, which manipulated frequency, duration, intensity, location, and gap. Haigh et al. (2023) used a roving pitch‐deviance design and did not report an autism–TD MMN latency outcome.

A smaller subset of studies reported autism‐associated latency shifts for specific non‐speech contrasts. Sato et al. (2025) reported shorter MMN latency in autism for a tone‐duration deviant (1000 Hz, 100 ms standard vs. 50 ms deviant), with effects observed at Fz and Cz. In contrast, Kasai et al. (2005) reported a left‐hemisphere prolongation in MMF latency in autistic adults compared to TD peers for a pure‐tone duration contrast (100 ms standard vs. 50 ms deviant). Kasai et al. (2005) further detected that MMF latency within the autism group was negatively correlated with neuroleptic and anticholinergic medication exposure, indicating pharmacological modulation of timing within autism.

#### 3.5.3 Mismatch Response Hemispheric Distribution

Adult non‐speech studies rarely tested hemispheric lateralization explicitly because most EEG analyses emphasized midline electrodes or region‐averaged measures without formal left–right statistical contrasts (Tables 3 and 6). Chien et al. (2018) quantified MMN exclusively at Fz for tone‐duration (1000 Hz, 50 ms vs. 100 ms) and tone‐frequency (1000 Hz vs. 1200 Hz) deviants, which precluded hemispheric comparison. Haigh et al. (2023) analyzed MMN in a roving pitch‐deviance paradigm using a dense frontocentral montage (F1, Fz, F2; FC1, FCz, FC2; C1, Cz, C2) and did not report hemisphere‐specific contrasts. Randeniya et al. (2022) reported MMN outcomes using Fz‐based analyses and whole‐scalp (64‐channel) measures for stochastic frequency deviants (500 Hz standards vs. 2000 Hz deviants), without lateralized testing. Sato et al. (2025) quantified MMN at Fz and Cz for a tone‐duration deviant (1000 Hz, 100 ms vs. 50 ms) and did not assess hemispheric asymmetry.

Some adult EEG studies recorded from bilateral electrode arrays, but hemispheric dominance was not systematically evaluated. Kujala et al. (2007) employed an extensive bilateral montage (F3/F4, Fz, C3/C4, Cz, T3/T4, Pt3/Pt4, M1/M2) to examine non‐speech deviants across multiple acoustic dimensions (frequency, duration, intensity, location, and gap). Although the study reported MMN effects at Fz and Cz for specific deviant types, it did not report an autism‐specific hemispheric lateralization pattern for non‐speech mismatch responses.

Whole‐head MEG provided the clearest hemisphere‐resolved evidence in adult non‐speech literature. Kasai et al. (2005) recorded MMF responses using a 122‐channel MEG system and localized activity to bilateral temporal regions. For a pure‐tone duration contrast (100 ms standard vs. 50 ms deviant), the study reported a left‐hemisphere prolongation of MMF latency in autistic adults relative to TD peers. This effect reflected a lateralized timing difference, rather than a generalized hemispheric dominance of mismatch responses, and MMF latency was further reported to correlate negatively with neuroleptic and anticholinergic medication exposure within the autism group.

Taken together, adult non‐speech studies did not support a consistent hemispheric lateralization pattern distinguishing autistic from TD adults. When hemisphere‐specific effects were observed, they were isolated, feature‐specific, and primarily reflected latency asymmetries detected in MEG studies, rather than robust amplitude lateralization across EEG paradigms.

### 3.6 Auditory MMN and MMF in Autistic Adults Using Speech Stimuli

#### 3.6.1 Mismatch Response Amplitude

Across adult speech paradigms, studies reported attenuated, enhanced, or no autism–TD differences in MMN/MMF amplitude, and the direction of the effect depended on the speech unit and on the feature that differed between the standard and deviant, including emotional prosody, pitch/fundamental frequency, and duration (Tables 3 and 6). Fan and Cheng (2014) used the nonsense syllable /dada/ spoken with neutral prosody as the standard and presented happy and angry prosody tokens as deviants. They reported smaller MMN amplitudes in autism than in TD adults and further showed that TD adults exhibited larger MMNs to angry than to happy deviants, whereas autistic adults did not show this emotional differentiation.

Kujala et al. (2005) used the Finnish word /Saara/ spoken with neutral prosody as the standard and presented deviants spoken with commanding, sad, and scornful prosody, which also differed in duration. The study reported attenuated MMN amplitude in autism relative to TD for specific prosodic deviants, with effects localized to right temporo‐parietal electrodes, while other prosodic contrasts did not yield a significant autism–TD amplitude difference.

Kujala et al. (2007) presented vowel stimuli (/a/ and /o/) and manipulated pitch/fundamental frequency and duration across standard–deviant contrasts. They reported larger MMN amplitudes in autism than TD for speech pitch deviants, with significant effects observed over right‐hemisphere, left‐hemisphere, and midline sites. The same study also reported a frontal MMN amplitude effect for speech duration deviants, indicating feature‐specific modulation rather than a uniform amplitude pattern.

For vowel‐identity contrasts measured with MEG, Matsuzaki et al. (2019) contrasted /a/ standards with /u/ deviants and reported no autism–TD difference in MMF power for this speech‐identity contrast. Fan and Cheng (2014) included Japanese speech conditions involving a duration change within /a/ and a phoneme identity contrast (/a/ vs. /o/), and observed longer MMF left‐hemisphere latencies in autism than in TD peers.

#### 3.6.2 Mismatch Response Latency

Latency findings for adult speech paradigms were limited and heterogeneous, with most studies reporting no autism–TD difference and a smaller subset indicating prolonged MMN/MMF latency in autism (Tables 3 and 6). Fan and Cheng (2014) examined emotional prosody using the syllable /dada/ and reported no between‐group difference in MMN latency for either happy or angry prosodic deviants. Kujala et al. (2005) similarly included speech prosody conditions using the word /Saara/ and did not report an autism–TD latency difference for the available prosodic contrasts.

In contrast, Kasai et al. (2005) provided evidence for prolonged MMF latency in autism, identifying a left‐hemisphere latency delay for at least one Japanese speech contrast involving either a duration change within /a/ or a phoneme identity contrast (/a/ vs. /o/). That study further reported that MMF latency within the autism group was negatively correlated with neuroleptic and anticholinergic medication exposure, indicating modulation of temporal processing by pharmacological agents. Matsuzaki et al. (2019) also reported prolonged MMF latency in autistic adults for a vowel‐identity contrast (/a/ standard vs. /u/ deviant), localized to the superior temporal gyrus.

#### 3.6.3 Mismatch Response Hemispheric Lateralization

Adult speech studies often constrained hemispheric inference because many EEG analyses emphasized midline electrodes or bilateral montages without formally testing left–right contrasts as primary outcomes. Fan and Cheng (2014) quantified MMN at Fz, Cz, and Pz while examining emotional prosody in the syllable /dada/ (neutral standard; happy and angry deviants). This approach supported autism–TD amplitude comparisons but did not permit conclusions regarding hemispheric dominance.

Several studies nevertheless reported hemisphere‐resolved effects for specific speech contrasts. Kujala et al. (2005) recorded from a bilateral temporo‐parietal montage (Fz, F3, CP3, CT3, TP3, F4, CP4, CT4, TP4, Cz) while examining emotional prosody and duration changes in the word /Saara/. The study reported an autism–TD difference localized to right temporo‐parietal electrodes for commanding and scornful prosody, whereas other prosodic contrasts did not yield lateralized group differences.

Lepistö et al. (2007) provided the most detailed EEG‐based hemispheric characterization in adult speech paradigms, using a broad bilateral montage (F3, C3, T3, TP3, Fz, Cz, Pz, F4, T4, P4, TP4). For speech pitch/fundamental frequency deviants applied to vowels /a/ and /o/, the study reported enhanced MMN amplitudes in autism across right‐hemisphere, left‐hemisphere, and midline sites. In contrast, speech duration deviants elicited a frontal MMN amplitude effect, indicating that hemispheric involvement varied by speech feature rather than reflecting a uniform lateralization pattern.

MEG studies contributed additional hemisphere‐specific information. Matsuzaki et al. (2019) localized MMF responses to the STG using a 275‐channel MEG system and reported right‐hemispheric lateralization of MMF power in autistic adults for a vowel‐identity contrast (/a/ standard vs. /u/ deviant). In contrast, Kasai et al. (2005) recorded MMF using a 122‐channel whole‐head MEG system, localized activity to bilateral temporal regions, and identified a left‐hemisphere prolongation of MMF latency in autism for at least one speech contrast, indicating that hemispheric effects in adults could emerge in timing rather than amplitude.

Taken together, the adult speech literature did not converge on a single lateralization pattern across paradigms. Instead, hemisphere‐resolved effects emerged in a stimulus‐ and feature‐specific manner, with right‐hemisphere involvement reported most consistently for emotional prosody and vowel‐based contrasts, alongside isolated left‐hemisphere latency effects identified in MEG studies.

### 3.7 Sex‐Related Considerations Across Studies

Across developmental stages, most studies combined data from male and female participants and frequently included predominantly male samples, reflecting—and potentially reinforcing—the higher proportion of males diagnosed with autism. Eight studies examined exclusively male cohorts (Ferri et al. 2003; Lindström et al. 2018; Ludlow et al. 2014; Mamashli et al. 2017; Matsuzaki et al. 2017, 2019; Sawada et al. 2008; Weismüller et al. 2015), further limiting representation of females across the literature.

Despite this imbalance, virtually no study conducted formal sex‐stratified analyses of MMN or MMF amplitude, latency, or hemispheric distribution in autistic participants. This absence was consistent across childhood, adolescence, and adulthood, including studies with sample sizes that might have supported exploratory sex‐based comparisons. The lack of sex‐specific analyses constrained the ability to characterize sex‐related auditory phenotypes and may have obscured biologically meaningful variability associated with sex.

### 3.8 Narrative Synthesis

Mismatch response amplitude showed age‐related variation, but patterns depended primarily on stimulus feature and context. In childhood, many non‐speech studies reported smaller MMN amplitudes in autistic children than in TD peers, most consistently for frequency contrasts and less consistently for duration contrasts, while other studies reported larger amplitudes or no clear group differences (Abdeltawwab and Baz 2015; Cary et al. 2024; Čeponiene et al. 2003; Di Lorenzo et al. 2020; Ferri et al. 2003; Gomot et al. 2011; Vlaskamp et al. 2017; Yu et al. 2015; Zhang et al. 2019). Age‐sensitive childhood analyses linked amplitude to age within autism and showed site‐dependent trajectories, with reduced group separation at frontal/central midline regions but persisting group differences at parietal midline regions (Abdeltawwab and Baz 2015; Ferri et al. 2003; Weismüller et al. 2015). Child speech paradigms similarly remained feature‐dependent, with smaller MMN amplitudes often reported for vowel/phoneme identity and emotional prosody, and larger amplitudes reported for some pitch/fundamental‐frequency contrasts (Lepistö et al. 2005, 2006, 2008; Lindström et al. 2016, 2018; Ruiz‐Martinez et al. 2020). In adolescence, studies reported smaller, larger, or comparable amplitudes across paradigms, and one sample showed smaller amplitudes in older than younger participants (Da Silva Mayerle et al. 2023; Hudac et al. 2018; Lassen et al. 2022). In adulthood, most non‐speech paradigms reported comparable MMN/MMF amplitudes between autistic and TD adults, with context‐ or feature‐specific deviations, and adult speech effects again depended on prosody‐ versus pitch‐related contrasts (Chien et al. 2018; Fan and Cheng 2014; Goris et al. 2018; Kujala et al. 2007; Lepistö et al. 2007; Matsuzaki et al. 2019; Randeniya et al. 2022).

This developmental pattern is also consistent with the most recent quantitative meta‐analysis, which reported an interaction between age group and design type, such that autistic children and adolescents showed reduced MMN amplitudes in multifeature paradigms, whereas autistic adults showed increased amplitudes under comparable contextual conditions (Sapey‐Triomphe et al. 2025) while substantial heterogeneity remained across stimulus features and designs.

Mismatch response latency remained heterogeneous across ages, with direction shaped by deviant type and participant characteristics. In childhood non‐speech paradigms, studies reported both earlier timing for frequency contrasts and longer timing for frequency‐, duration‐, or temporal‐structure deviants, while others reported comparable latencies despite amplitude differences (Abdeltawwab and Baz 2015; Di Lorenzo et al. 2020; Gomot et al. 2002; Huang et al. 2018; Sanglakh Ghoochan Atigh et al. 2024; Vlaskamp et al. 2017). One dataset suggested developmental moderation within childhood by showing shorter latency for duration deviants in a younger autistic subgroup (Schall et al. 2024). Across speech paradigms and MEG studies, latency differences often aligned with language profile, auditory sensitivity, or intolerance to change rather than appearing uniformly across autistic cohorts (Berman et al. 2016; Gomot et al. 2011; Green et al. 2020; Matsuzaki et al. 2017; Roberts et al. 2011).

Adolescent latency findings in autism remained limited and were often comparable to TD peers, although one study reported longer latency to a tone‐burst frequency contrast in autistic participants (Da Silva Mayerle et al. 2023; Lassen et al. 2022). In adulthood, MMN/MMF latencies in autism were generally similar to TD groups, with selective deviations including shorter latency to a duration deviant and left‐hemisphere timing differences in MEG. Speech‐evoked findings in adults were mixed, with null results for prosody contrasts but MEG evidence of longer latencies to vowel identity and other speech contrasts in autistic participants (Fan and Cheng 2014; Kasai et al. 2005; Matsuzaki et al. 2019; Sato et al. 2025).

Notably, although individual studies across childhood, adolescence, and adulthood reported longer or shorter mismatch latencies in autism for specific deviants or subgroups, the most recent quantitative meta‐analysis found no significant overall pooled autism–TD difference in MMN latency across age groups, and latency moderators, including age, did not significantly explain heterogeneity (Sapey‐Triomphe et al. 2025). This pattern is consistent with substantial cross‐study variability and limited statistical power for subgroup‐specific latency effects in the primary literature.

Hemispheric distribution provided the least developmentally resolved evidence in autism, partly because many EEG studies emphasized frontal or central midline quantification and many MEG studies reported source localization without formal hemispheric contrasts. In childhood, non‐speech paradigms yielded cohort‐ and analysis‐dependent patterns rather than a consistent lateralization profile relative to TD peers (Gomot et al. 2002, 2011; Lepistö et al. 2009). Speech paradigms in autistic children more often allowed inference of laterality and most frequently reported relatively stronger right‐hemisphere weighting for phoneme identity, pitch/fundamental frequency, emotional prosody, and lexical stress compared with TD groups (Jansson‐Verkasalo et al. 2003; Korpilahti et al. 2007; Lepistö et al. 2006; Zhang et al. 2018). In adolescence, MEG studies reported right‐lateralized mismatch responses in one complex‐tone paradigm and contralateral organization in another, again relative to TD peers (Mamashli et al. 2017; Tecchio et al. 2003). In adulthood, lateralization patterns remained stimulus‐ and measure‐specific in autism, including right temporo‐parietal effects for some prosody contrasts, right‐lateralized power for vowel identity, and left‐sided timing differences in MEG (Kasai et al. 2005; Kujala et al. 2005; Matsuzaki et al. 2019).

### 3.9 Quality Assessment

Study quality, as assessed using the Newcastle–Ottawa Scale, ranged from satisfactory (Matsuba et al. 2024; Sato et al. 2025) to good (Da Silva Mayerle et al. 2023; Green et al. 2020; Haigh et al. 2023; Hudac et al. 2018; Kabil et al. 2023; Lassen et al. 2022; Randeniya et al. 2022; Sanglakh Ghoochan Atigh et al. 2024; Schall et al. 2024; Zhang et al. 2019), with the majority rated as fair (Table S1). All studies included age‐matched control groups and used appropriate statistical approaches; however, few reported effect sizes or confidence intervals, limiting comparability across studies and constraining synthesis of magnitude and precision.

## 4 Discussion

We synthesized evidence from 55 passive oddball MMN/MMF studies comparing autistic individuals and TD peers across childhood, adolescence, and adulthood. We followed SWiM guidelines because the literature varied substantially in participant age, stimulus contrasts, recording modality (EEG vs. MEG), and analytic strategy (Campbell et al. 2020). This heterogeneity precluded quantitative pooling, so we compared boundary conditions qualitatively across development.

Across development, three patterns recurred in autistic groups compared with TD peers under specific conditions: smaller MMN/MMF amplitudes were reported most often in childhood; longer MMN/MMF latency emerged in a subset of studies, often for selected deviants and within‐autism subgroups; and when analyses permitted laterality inference, studies more often described relatively greater right‐hemisphere weighting, most clearly in speech paradigms. We interpreted these tendencies cautiously, as several datasets reported comparable or larger responses in autistic groups and many studies did not test hemispheric contrasts directly. Accordingly, we focused on what these boundary conditions implied biologically about auditory change detection in autism—how auditory systems formed and maintained regularity representations, how they timed deviance detection once regularities were established, and how speech‐relevant computations were distributed across hemispheres (Garrido et al. 2009; Näätänen et al. 2001, 2007, 2012).

### 4.1 Attenuated Mismatch Response Amplitude in Autism Across Development

When studies reported smaller MMN/MMF responses in autism, we interpreted this as consistent with reduced mismatch magnitude under those stimulus statistics, rather than as evidence for a uniform reduction in auditory deviance detection. MMN/MMF amplitude is commonly interpreted as reflecting how robustly auditory systems represent established regularities and how distinct deviants violate those regularities under repetitive stimulation.

Classical MMN accounts described mismatch amplitude as an index of automatic deviance detection that depended on building and briefly maintaining a representation of stimulus regularities against which new input was compared (Garrido et al. 2009; Näätänen et al. 2001, 2007). Within this framework, smaller mismatch responses reported in some autistic cohorts plausibly reflected weaker or less consistently expressed regularity representations, reduced deviant–regularity contrast at the neural level, or more rapid adjustment of the regularity representation that diminished the mismatch signal under particular stimulus statistics (Näätänen et al. 2012).

These ideas were consistent with the developmental boundary conditions observed across studies. Attenuated MMN/MMF amplitudes were reported most often in childhood—particularly in non‐speech paradigms and most consistently for frequency deviants—whereas findings in adolescence and adulthood were more mixed and depended on deviant feature and paradigm structure (Abdeltawwab and Baz 2015; Chien et al. 2018; Di Lorenzo et al. 2020; Gomot et al. 2011; Randeniya et al. 2022; Sanglakh Ghoochan Atigh et al. 2024; Sato et al. 2025; Vlaskamp et al. 2017). Age‐focused analyses within autistic child samples did not support a single monotonic trajectory; instead, they indicated selective modulation that varied by feature and scalp distribution, consistent with non‐uniform maturation of mismatch generators and their expression in EEG/MEG measures (Abdeltawwab and Baz 2015; Ferri et al. 2003; Weismüller et al. 2015). Together, these constraints supported an interpretation in which between‐group amplitude differences reflected interactions between developmental stage and the statistical demands of the paradigm, rather than a uniform lifespan phenotype in autism.

Speech paradigms strengthened the biological relevance of amplitude effects because they probed contrasts directly supporting speech perception. Several studies reported smaller mismatch responses for phoneme or vowel identity and emotional prosody deviants, extending attenuation beyond pure tones and implicating speech‐relevant regularity formation in at least a subset of cohorts (Fan and Cheng 2014; Korpilahti et al. 2007; Kujala et al. 2005; Lindström et al. 2016; Ludlow et al. 2014; Ruiz‐Martinez et al. 2020; Schall et al. 2024). In contrast, pitch‐based speech contrasts sometimes produced preserved or enhanced mismatch responses, indicating that the encoded acoustic feature and contextual structure constrained whether attenuation emerged (Lepistö et al. 2005, 2006, 2007, 2008). We therefore treated attenuated MMN/MMF amplitude as a recurring—but not universal—feature of auditory processing in autism, whose interpretability depended on developmental stage, deviant type, and stimulus statistics which may serve as a general marker of auditory or language outcomes (Näätänen et al. 2012).

### 4.2 Prolonged Mismatch Response Latency in Autism Across Development

We treated MMN/MMF latency as mechanistically informative because it constrained the timing of deviance detection once the auditory system established a regularity. Within sensory‐memory accounts, latency indexed how rapidly incoming input was compared with the current regularity representation and how quickly that representation was adjusted following a violation (Garrido et al. 2009; Näätänen et al. 2001, 2007). Under this framing, longer mismatch timing reported in some studies was compatible with slower deviance detection or slower regularity adjustment under particular stimulus statistics, without implying a uniform latency phenotype across autism. Consistent with this heterogeneity, the most recent quantitative meta‐analysis reported no overall autism–TD difference in MMN latency, indicating that timing effects are likely context‐ and subgroup‐dependent rather than a robust group‐level signature (Sapey‐Triomphe et al. 2025).

In childhood, several non‐speech paradigms reported longer MMN/MMF latencies for frequency, duration, or timing‐related deviants, whereas other studies reported no difference or earlier mismatch timing, including earlier timing for a tone‐frequency contrast (Abdeltawwab and Baz 2015; Čeponiene et al. 2003; Da Silva Mayerle et al. 2023; Di Lorenzo et al. 2020; Gomot et al. 2002; Huang et al. 2018; Sanglakh Ghoochan Atigh et al. 2024; Vlaskamp et al. 2017). Speech paradigms in children likewise showed mixed timing patterns, with longer latencies reported in some cohorts described at the time as autism for syllable, phoneme, and fundamental‐frequency contrasts, alongside null or shorter‐latency findings in other datasets (Jansson‐Verkasalo et al. 2003, 2005; Kemner et al. 1995; Matsuba et al. 2024; Weismüller et al. 2015).

Several studies suggested that timing effects were more strongly related to within‐autism variation than to diagnostic grouping alone. MEG speech studies reported longer MMF latency most clearly in autistic participants with more pronounced language challenges than in autistic peers with less pronounced language challenges and TD participants, whereas another dataset reported earlier MMN latency for a vowel contrast specifically in a language‐stratified autistic subgroup (Berman et al. 2016; Green et al. 2020; Roberts et al. 2011). Other studies related mismatch timing to auditory sensitivity profiles, and some studies linked shorter latency to higher intolerance to change, again emphasizing phenotype dependence (Gomot et al. 2011; Matsuzaki et al. 2017). Schall et al. (2024) further supported age‐ and deviant‐dependent timing within childhood by reporting shorter latency for duration deviants specifically in a younger autistic subgroup.

Evidence in adolescence and adulthood did not support a uniform developmental pattern. In adolescence, latency differences were often absent, with prolonged timing reported only in specific paradigms (Da Silva Mayerle et al. 2023; Lassen et al. 2022; Mamashli et al. 2017). In adulthood, most non‐speech paradigms reported comparable timing, with exceptions in both directions and hemisphere‐specific timing differences reported in MEG (Chien et al. 2018; Kasai et al. 2005; Kujala et al. 2007; Randeniya et al. 2022; Sato et al. 2025). Adult speech studies likewise showed mixed outcomes, including largely null latency effects in prosody paradigms and longer MMF latency for vowel identity contrasts in MEG (Fan and Cheng 2014; Kujala et al. 2005; Matsuzaki et al. 2019).

Taken together, prolonged latency emerged as a recurrent but non‐universal feature whose interpretability depended on developmental stage, deviant feature, and phenotype. When timing differences appeared in speech paradigms and language‐stratified analyses, they plausibly reflected reduced efficiency in processing rapidly changing acoustic cues that support phoneme identification and higher‐order speech computations, while still requiring caution because language‐stratified subgroups also showed earlier timing under some contrasts (Berman et al. 2016; Garrido et al. 2009; Green et al. 2020; Näätänen et al. 2007; Roberts et al. 2011).

### 4.3 Auditory Mismatch Responses to Speech Stimuli in Autism Across Development

We interpreted speech‐evoked MMN/MMF findings as evidence for feature‐ and phenotype‐dependent differences in how autistic samples encoded linguistically relevant regularities, rather than evidence for a single direction of altered speech discrimination. In childhood, several studies reported attenuated mismatch amplitudes for phoneme or vowel identity and prosodic meaning contrasts, while other studies reported null effects for specific contrasts or failed to elicit a reliable mismatch response in either group, limiting inference (Kemner et al. 1995; Korpilahti et al. 2007; Kujala et al. 2010; Lindström et al. 2016, 2018; Matsuba et al. 2024; Ruiz‐Martinez et al. 2020; Schall et al. 2024; Weismüller et al. 2015). Several datasets also reported enhanced mismatch amplitudes for pitch or fundamental‐frequency manipulations in particular cohorts and acoustic contexts, while other contrasts within the same studies yielded reduced or absent effects, underscoring feature dependence (Jansson‐Verkasalo et al. 2005; Lepistö et al. 2005, 2006, 2008). Speech evidence in adolescence remained sparse, with one study reporting reduced mismatch amplitude for word and pseudoword deviants (Ludlow et al. 2014). In adulthood, prosody paradigms more often reported attenuated mismatch amplitude for emotionally salient deviants, whereas a pitch‐based vowel study reported larger mismatch amplitudes and MEG vowel‐identity work reported comparable power alongside prolonged timing (Fan and Cheng 2014; Kujala et al. 2005; Lepistö et al. 2007; Matsuzaki et al. 2019).

These mixed patterns supported multi‐level interpretation rather than a single explanatory model. The *Allophonic Perception Theory of Autism* proposed unusually fine‐grained acoustic sensitivity alongside reduced stability of phoneme‐level category representations, a profile compatible with attenuated mismatch for contrasts carrying clear linguistic or socio‐communicative meaning in a subset of samples (You et al. 2017). The *Weak Central Coherence* framework offered a complementary account by positing a bias toward local feature processing that could preserve sensitivity to particular acoustic cues while constraining integration into higher‐order linguistic representations (Happé and Frith 2006). Broader work on language development and semantic and lexical integration supported viewing speech perception within a wider cognitive–linguistic phenotype rather than as a unitary auditory difference (Arunachalam and Luyster 2016; Bebko et al. 2014; DePape et al. 2012; Stewart et al. 2018).

Anchoring these findings to the physiology indexed by MMN/MMF further clarified their interpretation. Classical accounts described MMN/MMF as reflecting the formation and short‐lived maintenance of stimulus regularities and the generation of a mismatch signal when those regularities were violated (Garrido et al. 2009; Näätänen et al. 2001, 2007). From this perspective, attenuated speech‐evoked mismatch amplitudes could reflect weaker or less consistently expressed regularity representations for linguistically meaningful contrasts, whereas enhanced responses to pitch‐based deviants could reflect relatively strong encoding of specific acoustic dimensions in particular contexts. Timing and laterality findings sharpened this account by emphasizing phenotype dependence and network organization: longer speech‐evoked MMF latency emerged most clearly in autistic participants with more pronounced language challenges in some MEG cohorts, while another dataset reported earlier MMN latency in a language‐stratified autistic subgroup (Berman et al. 2016; Green et al. 2020; Roberts et al. 2011). When analyses permitted laterality inference, studies more often described relatively greater right‐hemisphere weighting for speech contrasts emphasizing phoneme identity, pitch/fundamental frequency, emotional prosody, or lexical stress, although many studies did not implement explicit hemispheric contrasts (Jansson‐Verkasalo et al. 2003; Korpilahti et al. 2007; Kujala et al. 2005; Lepistö et al. 2006; Matsuzaki et al. 2019; Zhang et al. 2018). Taken together, the speech literature supported a developmental account in which MMN/MMF group differences reflected interacting influences across stimulus acoustics, regularity formation/sensory‐memory demands, temporal dynamics, hemispheric weighting, and language and sensory profiles rather than a single uniform alteration in speech discrimination across autism (Näätänen et al. 2012).

### 4.4 Right‐Hemispheric Distribution of Auditory Mismatch Responses in Autism Across Development

We interpreted laterality findings as suggestive but constrained because many EEG studies quantified mismatch using frontal or central midline measures or region‐averaged approaches that did not permit formal left–right comparisons, even when group differences in amplitude or latency were reported. Some studies further limited inference by quantifying mismatch at a single lateral site or by providing insufficient montage detail. Consequently, hemisphere‐resolved inference relied primarily on the subset of studies that explicitly tested hemispheric weighting or used MEG source localization with hemisphere‐resolved reporting.

Within childhood, speech paradigms provided the clearest evidence for altered hemispheric weighting when laterality was testable. Studies more often described relatively stronger right‐hemisphere involvement for speech contrasts emphasizing phoneme identity, pitch/fundamental frequency, emotional prosody, or lexical stress, and some reports described a redistribution—right‐weighting alongside reduced left‐weighting—rather than a uniform change in response magnitude (Jansson‐Verkasalo et al. 2003; Korpilahti et al. 2007; Lepistö et al. 2006; Zhang et al. 2018). In contrast, child non‐speech paradigms rarely enabled robust lateralization tests and yielded cohort‐ and analysis‐dependent patterns, including null distribution differences in some broad‐montage studies and site‐specific effects in others (Gomot et al. 2002, 2011; Lepistö et al. 2009). In adolescence, EEG studies again tended to emphasize midline measures, whereas MEG provided clearer hemisphere‐relevant evidence by reporting right‐lateralized mismatch responses for a complex‐tone paradigm and contralateral auditory organization in another MEG study (Mamashli et al. 2017; Tecchio et al. 2003). In adulthood, EEG and MEG findings suggest that hemispheric effects could emerge in mismatch magnitude or timing, including right‐weighted distributions for prosody‐related effects and hemisphere‐specific latency prolongation in MEG for at least one contrast (Kasai et al. 2005; Kujala et al. 2005; Matsuzaki et al. 2019). Collectively, these constraints did not support a single, developmentally stable right‐lateralization signature; instead, they pointed to feature‐ and method‐dependent hemispheric weighting, with the most repeatable right‐weighted patterns emerging for speech contrasts relying strongly on prosody, stress, or pitch cues.

We interpreted these mismatch laterality observations within broader neurobiological work showing differences in hemispheric specialization and interhemispheric organization in language‐relevant systems in autism. Structural and functional neuroimaging studies reported altered asymmetry and lateralization in language‐associated regions, and connectivity studies described differences in intra‐ and interhemispheric coordination that related to variability in language and communication (Flagg et al. 2005; Floris et al. 2016; Herbert et al. 2002, 2005; Herringshaw et al. 2016). Developmental studies further suggest that lateralization and tract‐level asymmetry could diverge early and relate to later language outcomes or autism‐related traits (Finch et al. 2017; Liu et al. 2019; Wei et al. 2018). However, because MMN/MMF studies rarely tested direct associations between mismatch laterality and continuous language measures, we treated right‐weighted mismatch involvement as mechanistically plausible—particularly for prosody and stress contrasts—rather than as a validated marker of language phenotype. Future work combining symmetric coverage, explicit laterality indices, continuous language and sensory measures, and integration with structural and functional connectivity metrics will be best positioned to determine whether mismatch laterality tracks meaningful variation in speech perception and contextual auditory processing across autism.

### 4.5 Linking Sensory‐Memory Accounts and Precision‐Weighted Predictive Processing

From sensory‐memory accounts, MMN/MMF indexed automatic deviance detection based on forming and briefly maintaining representations of auditory regularities (Näätänen et al. 2001, 2007). Predictive‐processing models offered a complementary computational description in which auditory systems inferred recent acoustic statistics, generated expectations, and produced mismatch responses when deviants violated those expectations (Clark 2013; Friston 2005; Garrido et al. 2009). In this framework, the sensory‐memory “trace” corresponded to an internal generative model of recent acoustic context, and MMN/MMF reflected prediction‐error signaling evoked by violations of that model. Precision weighting set the gain on prediction errors relative to the stability of current expectations (Friston 2005; Garrido et al. 2009; Hohwy 2013).

Attenuated MMN/MMF amplitude in autistic groups relative to TD peers was compatible with weaker neural expression of deviance when the inferred regularity was represented with lower precision or reduced stability. Under attenuated‐prior (*hypo‐prior*) proposals, reduced precision of prior expectations meant that deviants violated a less confident regularity representation, yielding smaller prediction errors and therefore smaller mismatch responses even when discrimination remained possible (Brock 2012; Pellicano and Burr 2012). Aberrant‐precision accounts offered a complementary route to attenuation by emphasizing context dependence: if prediction‐error gain and updating operated differently under particular stimulus statistics, the internal model could adjust quickly after violations, reducing the sustained deviant–regularity contrast that contributed to MMN/MMF magnitude (Hohwy 2013; Van de Cruys et al. 2013). This framing therefore predicted selective attenuation, emerging most clearly in paradigms that demanded stable extraction and maintenance of regularities, rather than a uniform signature across autism (Näätänen et al. 2012).

Predictive processing also framed prolonged MMN/MMF latency by emphasizing the time required to establish that an event was unlikely under the current model and to revise that model after a violation. When regularity representations were uncertain or when prediction errors were effectively down‐weighted for the relevant deviant dimension, the system could require more evidence to confirm a violation, producing later mismatch peaks without implying global slowing of auditory processing (Clark 2013; Friston 2005; Garrido et al. 2009; Hohwy 2013). The same architecture also accommodated the mixed and sometimes null findings in this review: under highly predictable statistics, autistic and TD groups could converge on similarly stable expectations, yielding comparable mismatch responses, whereas changes in predictability, deviant salience, and phenotype could shift effects toward attenuation, enhancement, or null outcomes. In particular, *High and Inflexible Precision of Prediction Errors in Autism* (HIPPEA) emphasized that atypical precision assignment could yield bidirectional effects across contexts by changing the balance between expectation strength and prediction‐error gain (Lawson et al. 2017; Näätänen et al. 2012; Van de Cruys et al. 2013, 2014).

### 4.6 Recommendations for Future Research

To clarify the developmental mechanisms underlying auditory mismatch processing in autism, future research should prioritize longitudinal designs that explicitly track age‐related changes in MMN and MMF amplitude, latency, and spatial distribution. Such designs are essential to distinguish true maturational trajectories from cohort effects and to determine whether apparent age‐related convergence or divergence reflects developmental change rather than methodological variability. Particular attention should be paid to sex as a moderating factor, given its near‐complete omission from formal analyses in the existing literature despite consistent male overrepresentation.

Future studies would also benefit from the use of ecologically valid and culturally adapted stimuli, including speech, emotional, and multisensory signals, to better approximate real‐world auditory environments. As shown across the reviewed studies, stimulus complexity and relevance strongly influenced mismatch responses; therefore, incorporating multimodal paradigms may help clarify how auditory prediction mechanisms interact with broader sensory and social processing differences indexed by MMN and MMF responses (Bathelt et al. 2017; Schwartz et al. 2018; Thye et al. 2018; Vlaskamp et al. 2017).

Methodological heterogeneity, including variability in electrode selection, time windows, deviant definitions, and analytic strategies, remains a major limitation of the field and constrains reproducibility and generalizability. Addressing this will require collaborative, multi‐site initiatives with sufficiently powered samples, preregistered analysis plans, and transparent reporting of effect sizes and confidence intervals (Button et al. 2013; Goodman et al. 2016; Ioannidis 2005). The development and adoption of consensus guidelines for MMN/MMF acquisition and analysis, including standardized time windows and reporting of spatial coverage, would substantially strengthen cross‐study comparability (Poldrack 2019; Steegen et al. 2016).

Autistic females remain markedly underrepresented in MMN and MMF research. Given evidence that current diagnostic frameworks and phenotypic definitions are male‐biased, future work should move beyond binary sex comparisons and adopt trait‐based and dimensional recruitment strategies to better capture sex‐related variability in auditory processing phenotypes (Lai et al. 2015; Rubenstein et al. 2015).

Finally, MMN and MMF research in autism remains disproportionately concentrated in high‐income countries. Expanding research efforts into low‐ and middle‐income settings, where autism prevalence is also substantial, will be critical for evaluating the cross‐cultural robustness and global generalizability of electrophysiological markers (Adak and Halder 2017; Bakare and Munir 2011; Hossain et al. 2017).

### 4.7 Study Strengths and Limitations

This systematic review applied broad inclusion criteria and study‐design–specific quality assessments, enabling the synthesis of a diverse body of MMN and MMF literature spanning childhood, adolescence, and adulthood, across both speech and non‐speech paradigms. Most included studies were rated as fair quality, with a subset rated as good and only two rated as satisfactory. All studies employed age‐matched control groups and appropriate statistical approaches for their respective designs.

A major strength of this review lies in its adherence to SWiM guidelines, which allowed for a structured, transparent, and theory‐informed synthesis in the context of substantial methodological heterogeneity. The use of three major bibliographic databases and explicit table‐driven cross‐checking ensured comprehensive coverage and reduced the risk of selective interpretation.

Nevertheless, several limitations should be acknowledged. Effect sizes and confidence intervals were inconsistently reported, limiting quantitative comparison across studies. Many investigations relied on small samples, midline electrode configurations, or analytic choices that precluded formal assessment of hemispheric lateralization, sex effects, or individual‐level variability. These constraints necessitated cautious interpretation and precluded strong mechanistic conclusions.

Overall, despite these limitations, the present synthesis provides a rigorous and conservative integration of the MMN/MMF literature in autism, highlighting robust patterns of heterogeneity, context dependence, and developmental modulation while clearly delineating the boundaries of current empirical support.

## 5 Conclusion

We synthesized 55 passive oddball MMN/MMF studies comparing autistic individuals with TD peers across development. Findings were heterogeneous, but three patterns recurred under specific conditions: attenuated mismatch responses appeared most often in childhood, prolonged mismatch timing emerged in a subset of paradigms and within‐autism subgroups, and studies permitting laterality inference more often suggest relatively greater right‐hemisphere weighting, especially for speech. Sensory‐memory and predictive‐processing accounts can accommodate both these tendencies and the many null or opposite‐direction results by emphasizing context‐ and phenotype‐dependent differences in how auditory systems form, maintain, and update inferred regularities. Future work should test these explanations with longitudinal, well‐powered designs that measure language and sensory profiles continuously, standardize reporting, and manipulate predictability to directly probe mechanisms underlying heterogeneity.

## Conflicts of Interest

The authors declare no conflicts of interest.

## Supporting information


**Table S1:** Quality assessment.

## Data Availability

Data sharing not applicable to this article as no datasets were generated or analysed during the current study.
