# Supplementary material for: Auditory Sensitivity in Autism: A Systematic Review of Mismatch Negativity and Mismatch Field Responses
Source: Autism Res. 2026 May 17;19(7):e70275. doi: 10.1002/aur.70275 (PMC13377376; doi:10.1002/aur.70275)
Supplement: Supplementary file 1 — Table S1: Quality assessment. [file AUR-19-0-s001.docx]

**Supporting Information**

**Search Strategy:** **Embase**

| 1 | (autis* adj1 disorder*).mp. [mp=title, abstract, heading word, drug trade name, original title, device manufacturer, drug manufacturer, device trade name, keyword, floating subheading word, candidate term word] |
| --- | --- |
| 2 | ASD.mp. [mp=title, abstract, heading word, drug trade name, original title, device manufacturer, drug manufacturer, device trade name, keyword, floating subheading word, candidate term word] |
| 3 | mismatch negativity. mp. [mp=title, abstract, heading word, drug trade name, original title, device manufacturer, drug manufacturer, device trade name, keyword, floating subheading word, candidate term word] |
| 4 | MMN.mp. [mp=title, abstract, heading word, drug trade name, original title, device manufacturer, drug manufacturer, device trade name, keyword, floating subheading word, candidate term word] |
| 5 | mismatch field. mp. [mp=title, abstract, heading word, drug trade name, original title, device manufacturer, drug manufacturer, device trade name, keyword, floating subheading word, candidate term word] |
| 6 | MMF.mp. [mp=title, abstract, heading word, drug trade name, original title, device manufacturer, drug manufacturer, device trade name, keyword, floating subheading word, candidate term word] |
| 7 | 1 or 2 |
| 8 | 3 or 4 |
| 9 | 5 or 6 |
| 10 | 7 and 8 |
| 11 | 7 and 9 |
| 12 | odd ball task*.mp. [mp=title, abstract, heading word, drug trade name, original title, device manufacturer, drug manufacturer, device trade name, keyword, floating subheading word, candidate term word] |
| 13 | odd ball paradigm*. mp. [mp=title, abstract, heading word, drug trade name, original title, device manufacturer, drug manufacturer, device trade name, keyword, floating subheading word, candidate term word] |
| 14 | 12 or 13 |
| 15 | 7 and 14 |
| 16 | audit* event related potential*.mp. [mp=title, abstract, heading word, drug trade name, original title, device manufacturer, drug manufacturer, device trade name, keyword, floating subheading word, candidate term word] |
| 17 | 7 and 16 |
| 18 | audit* ERP.mp. [mp=title, abstract, heading word, drug trade name, original title, device manufacturer, drug manufacturer, device trade name, keyword, floating subheading word, candidate term word] |
| 19 | 7 and 18 |
| 20 | electroencephalogram.mp. [mp=title, abstract, heading word, drug trade name, original title, device manufacturer, drug manufacturer, device trade name, keyword, floating subheading word, candidate term word] |
| 21 | limit 20 to english language |
| 22 | EEG.mp. [mp=title, abstract, heading word, drug trade name, original title, device manufacturer, drug manufacturer, device trade name, keyword, floating subheading word, candidate term word] |
| 23 | limit 22 to english language |
| 24 | 21 or 23 |
| 25 | 7 and 24 |
| 26 | 8 and 25 |
| 27 | magnetoencephalography.mp. [mp=title, abstract, heading word, drug trade name, original title, device manufacturer, drug manufacturer, device trade name, keyword, floating subheading word, candidate term word] |
| 28 | MEG.mp. [mp=title, abstract, heading word, drug trade name, original title, device manufacturer, drug manufacturer, device trade name, keyword, floating subheading word, candidate term word] |
| 29 | 27 or 28 |
| 30 | 7 and 29 |
| 31 | 9 and 30 |
| 32 | audit* discrimin*.mp. [mp=title, abstract, heading word, drug trade name, original title, device manufacturer, drug manufacturer, device trade name, keyword, floating subheading word, candidate term word] |
| 33 | 7 and 32 |
| 34 | audit* event related field.mp. [mp=title, abstract, heading word, drug trade name, original title, device manufacturer, drug manufacturer, device trade name, keyword, floating subheading word, candidate term word] |
| 35 | audit* ERF.mp. [mp=title, abstract, heading word, drug trade name, original title, device manufacturer, drug manufacturer, device trade name, keyword, floating subheading word, candidate term word] |
| 36 | 34 or 35 |
| 37 | 7 and 36 |
| 38 | asperger*.mp. [mp=title, abstract, heading word, drug trade name, original title, device manufacturer, drug manufacturer, device trade name, keyword, floating subheading word, candidate term word] |
| 39 | 8 and 38 |
| 40 | 9 and 38 |
| 41 | 14 and 38 |
| 42 | 16 and 38 |
| 43 | 18 and 38 |
| 44 | 29 and 38 |
| 45 | 32 and 38 |
| 46 | 36 and 38 |
| 47 | oddball task*.mp. [mp=title, abstract, heading word, drug trade name, original title, device manufacturer, drug manufacturer, device trade name, keyword, floating subheading word, candidate term word] |
| 48 | oddball paradigm*.mp. [mp=title, abstract, heading word, drug trade name, original title, device manufacturer, drug manufacturer, device trade name, keyword, floating subheading word, candidate term word] |
| 49 | 47 or 48 |
| 50 | 7 and 49 |
| 51 | 38 and 49 |
| 52 | cortical discrim*. mp. [mp=title, abstract, heading word, drug trade name, original title, device manufacturer, drug manufacturer, device trade name, keyword, floating subheading word, candidate term word] |
| 53 | 7 and 52 |
| 54 | 38 and 52 |
| 55 | sound discrim*.mp. [mp=title, abstract, heading word, drug trade name, original title, device manufacturer, drug manufacturer, device trade name, keyword, floating subheading word, candidate term word] |
| 56 | 7 and 55 |
| 57 | pervasive developmental disorder*.mp. [mp=title, abstract, heading word, drug trade name, original title, device manufacturer, drug manufacturer, device trade name, keyword, floating subheading word, candidate term word] |
| 58 | PDD.mp. [mp=title, abstract, heading word, drug trade name, original title, device manufacturer, drug manufacturer, device trade name, keyword, floating subheading word, candidate term word] |
| 59 | 57 or 58 |
| 60 | 8 and 59 |
| 61 | 9 and 59 |
| 62 | 16 and 59 |
| 63 | 32 or 52 or 55 |
| 64 | 29 and 59 |
| 65 | 32 or 52 or 55 |
| 66 | 59 and 65 |
| 67 | 36 and 59 |
| 68 | 49 and 59 |
| 69 | 8 and 24 and 38 |
| 70 | 9 and 29 and 38 |
| 71 | 8 and 24 and 59 |
| 72 | 9 and 29 and 59 |
| 73 | PDD-NOS.mp. [mp=title, abstract, heading word, drug trade name, original title, device manufacturer, drug manufacturer, device trade name, keyword, floating subheading word, candidate term word] |
| 74 | (pervasive developmental disorder- not otherwise specified).mp. [mp=title, abstract, heading word, drug trade name, original title, device manufacturer, drug manufacturer, device trade name, keyword, floating subheading word, candidate term word] |
| 75 | 73 or 74 |
| 76 | 8 and 75 |
| 77 | 9 and 75 |
| 78 | 49 and 75 |
| 79 | 16 and 75 |
| 80 | 18 and 75 |
| 81 | 36 and 75 |
| 82 | 65 and 75 |
| 83 | 8 and 24 and 75 |
| 84 | 9 and 29 and 75 |
| 85 | childhood disintegrative disorder.mp. [mp=title, abstract, heading word, drug trade name, original title, device manufacturer, drug manufacturer, device trade name, keyword, floating subheading word, candidate term word] |
| 86 | CDD.mp. [mp=title, abstract, heading word, drug trade name, original title, device manufacturer, drug manufacturer, device trade name, keyword, floating subheading word, candidate term word] |
| 87 | Heller's syndrome. mp. [mp=title, abstract, heading word, drug trade name, original title, device manufacturer, drug manufacturer, device trade name, keyword, floating subheading word, candidate term word] |
| 88 | 85 or 86 or 87 |
| 89 | 8 and 88 |
| 90 | 9 and 88 |
| 91 | 49 and 88 |
| 92 | 16 or 18 |
| 93 | 88 and 92 |
| 94 | 36 and 88 |
| 95 | 65 and 88 |

**Search Strategy:** **Medline**

| 1 | (autis* adj2 disorder*).mp. [mp=title, abstract, original title, name of substance word, subject heading word, floating sub-heading word, keyword heading word, organism supplementary concept word, protocol supplementary concept word, rare disease supplementary concept word, unique identifier, synonyms] |
| --- | --- |
| 2 | exp Autism Spectrum Disorder/ or exp Autistic Disorder/ or ASD.mp. |
| 3 | 1 or 2 |
| 4 | Evoked Potentials, Auditory/ or mismatch negativity.mp. |
| 5 | MMN.mp. |
| 6 | 4 or 5 |
| 7 | 3 and 6 |
| 8 | Evoked Potentials, Auditory/ or mismatch field.mp. |
| 9 | MMF.mp. [mp=title, abstract, original title, name of substance word, subject heading word, floating sub-heading word, keyword heading word, organism supplementary concept word, protocol supplementary concept word, rare disease supplementary concept word, unique identifier, synonyms] |
| 10 | 8 or 9 |
| 11 | 3 and 10 |
| 12 | Evoked Potentials/ and oddball task*.mp. |
| 13 | 3 and 12 |
| 14 | oddball paradigm*.mp. and exp Evoked Potentials, Auditory/ |
| 15 | 3 and 14 |
| 16 | audit* event related potential*.mp. |
| 17 | audit* ERP*.mp. |
| 18 | 16 or 17 |
| 19 | 3 and 18 |
| 20 | audit* event-related field*.mp. [mp=title, abstract, original title, name of substance word, subject heading word, floating sub-heading word, keyword heading word, organism supplementary concept word, protocol supplementary concept word, rare disease supplementary concept word, unique identifier, synonyms] |
| 21 | 3 and 20 |
| 22 | 22 audit ERF*.mp. [mp=title, abstract, original title, name of substance word, subject heading word, floating sub-heading word, keyword heading word, organism supplementary concept word, protocol supplementary concept word, rare disease supplementary concept word, unique identifier, synonyms] |
| 23 | exp Asperger Syndrome/ |
| 24 | 6 and 23 |
| 25 | 10 and 23 |
| 26 | 12 and 23 |
| 27 | 14 and 23 |
| 28 | 18 and 23 |
| 29 | 20 and 23 |
| 30 | 22 and 23 |
| 31 | audit* discrim*. mp. |
| 32 | sound* discrim*. mp. |
| 33 | cortical discrim*.mp. [mp=title, abstract, original title, name of substance word, subject heading word, floating sub-heading word, keyword heading word, organism supplementary concept word, protocol supplementary concept word, rare disease supplementary concept word, unique identifier, synonyms] |
| 34 | 31 or 32 or 33 |
| 35 | 3 and 34 |
| 36 | 23 and 34 |
| 37 | pervasive developmental disorder*.mp. |
| 38 | PDD.mp. or Child Development Disorders, Pervasive/ |
| 39 | 37 or 38 |
| 40 | 6 and 39 |
| 41 | 10 and 39 |
| 42 | 12 and 39 |
| 43 | 14 and 39 |
| 44 | 18 and 39 |
| 45 | 20 and 39 |
| 46 | 34 and 39 |
| 47 | Child Development Disorders, Pervasive/ or pervasive developmental disorder-not otherwise specified.mp. |
| 48 | exp Child Development Disorders, Pervasive/ or PDD-NOS.mp. |
| 49 | 47 or 48 |
| 50 | 6 and 49 |
| 51 | 10 and 49 |
| 52 | 12 and 49 |
| 53 | 14 and 49 |
| 54 | 18 and 49 |
| 55 | 20 and 49 |
| 56 | 34 and 49 |
| 57 | childhood disintegrative disorder.mp. |
| 58 | CDD.mp. |
| 59 | Heller's syndrome. mp. |
| 60 | 57 or 58 or 59 |
| 61 | 6 and 60 |
| 62 | 10 and 60 |
| 63 | 12 and 60 |
| 64 | 14 and 60 |
| 65 | 18 and 60 |
| 66 | 20 and 60 |
| 67 | 34 and 60 |
| 68 | Magnetoencephalography/ or MEG.mp. |
| 69 | 10 and 68 |
| 70 | 3 and 69 |
| 71 | 23 and 69 |
| 72 | 39 and 69 |
| 73 | 49 and 69 |
| 74 | 60 and 69 |
| 75 | EEG.mp. or Electroencephalography/ |
| 76 | 6 and 75 |
| 77 | 3 and 76 |
| 78 | 23 and 76 |
| 79 | 39 and 76 |
| 80 | 49 and 76 |
| 81 | 60 and 76 |

**Search Strategy:** **PsycInfo**

| 1 | Mismatch Negativity/ |
| --- | --- |
| 2 | MMN.mp. |
| 3 | 1 or 2 |
| 4 | mismatch field. mp. |
| 5 | MMF.mp. |
| 6 | 4 or 5 |
| 7 | (autis* adj2 disorder*).mp. [mp=title, abstract, heading word, table of contents, key concepts, original title, tests & measures, mesh] |
| 8 | 3 and 7 |
| 9 | 6 and 7 |
| 10 | exp Auditory Evoked Potentials/ and oddball task*.mp. |
| 11 | exp Auditory Evoked Potentials/ and oddball paradigm*.mp. |
| 12 | 10 or 11 |
| 13 | 7 and 12 |
| 14 | audit* event-related potential*.mp. and Electroencephalography/ |
| 15 | audit* ERP.mp. |
| 16 | 14 or 15 |
| 17 | 7 and 16 |
| 18 | audit* event-related field*.mp. [mp=title, abstract, heading word, table of contents, key concepts, original title, tests & measures, mesh] |
| 19 | audit* ERF.mp. [mp=title, abstract, heading word, table of contents, key concepts, original title, tests & measures, mesh] |
| 20 | 18 or 19 |
| 21 | 7 and 20 |
| 22 | Magnetoencephalography/ |
| 23 | 6 and 22 |
| 24 | 7 and 23 |
| 25 | audit* discrim*.mp. [mp=title, abstract, heading word, table of contents, key concepts, original title, tests & measures, mesh] |
| 26 | sound discrim*.mp. [mp=title, abstract, heading word, table of contents, key concepts, original title, tests & measures, mesh] |
| 27 | cortical discrim*.mp. [mp=title, abstract, heading word, table of contents, key concepts, original title, tests & measures, mesh] |
| 28 | 25 or 26 or 27 |
| 29 | 7 and 28 |
| 30 | ASD.mp. [mp=title, abstract, heading word, table of contents, key concepts, original title, tests & measures, mesh] |
| 31 | 3 and 30 |
| 32 | 6 and 30 |
| 33 | 12 and 30 |
| 34 | 16 and 30 |
| 35 | 20 and 30 |
| 36 | 23 and 30 |
| 37 | 28 and 30 |
| 38 | Autism Spectrum Disorders/ or asperger.mp. |
| 39 | 3 and 38 |
| 40 | 6 and 38 |
| 41 | 12 and 38 |
| 42 | 16 and 38 |
| 43 | 20 and 38 |
| 44 | 23 and 38 |
| 45 | 28 and 38 |
| 46 | Autism Spectrum Disorders/ or pervasive developmental disorder*.mp. |
| 47 | PDD.mp. [mp=title, abstract, heading word, table of contents, key concepts, original title, tests & measures, mesh] |
| 48 | 46 or 47 |
| 49 | 3 and 48 |
| 50 | 6 and 48 |
| 51 | 12 and 48 |
| 52 | 16 and 48 |
| 53 | 20 and 48 |
| 54 | 23 and 48 |
| 55 | 28 and 48 |
| 56 | Autism Spectrum Disorders/ or pervasive developmental disorder-not otherwise specified.mp. |
| 57 | "PDD-NOS".mp. [mp=title, abstract, heading word, table of contents, key concepts, original title, tests & measures, mesh] |
| 58 | 56 or 57 |
| 59 | 3 and 58 |
| 60 | 6 and 58 |
| 61 | 12 and 58 |
| 62 | 16 and 58 |
| 63 | 20 and 58 |
| 64 | 23 and 58 |
| 65 | 28 and 58 |
| 66 | "childhood disintegrative disorder".mp. [mp=title, abstract, heading word, table of contents, key concepts, original title, tests & measures, mesh] |
| 67 | CDD.mp. [mp=title, abstract, heading word, table of contents, key concepts, original title, tests & measures, mesh] |
| 68 | "heller's syndrome".mp. [mp=title, abstract, heading word, table of contents, key concepts, original title, tests & measures, mesh] |
| 69 | 66 or 67 or 68 |
| 70 | 3 and 69 |
| 71 | 6 and 69 |
| 72 | 12 and 69 |
| 73 | 16 and 69 |
| 74 | 20 and 69 |
| 75 | 23 and 69 |
| 76 | 28 and 69 |

**Supporting Table 1. Quality Assessment**

| **Study** | **Selection** | **Comparability** | **Outcome** | **Total Score** | **Methodological Quality** |
| --- | --- | --- | --- | --- | --- |
| Abdeltawwab and Baz (2015) | 3 | 2 | 2 | 7 | Fair |
| Berman et al. (2016) | 3 | 2 | 2 | 7 | Fair |
| Cary et al. (2024) | 4 | 1 | 2 | 7 | Fair |
| Čeponienė et al. (2003) | 3 | 1 | 2 | 6 | Fair |
| Charpentier et al. (2018) | 3 | 1 | 2 | 6 | Fair |
| Chien et al. (2018) | 3 | 2 | 2 | 7 | Fair |
| Da Silva Mayerle et al. (2023) | 4 | 1 | 2 | 7 | Good |
| Di Lorenzo et al. (2020) | 3 | 2 | 2 | 7 | Fair |
| Fan and Cheng (2014) | 3 | 2 | 2 | 7 | Fair |
| Ferri et al. (2003) | 3 | 1 | 2 | 6 | Fair |
| Gomot et al. (2002) | 3 | 2 | 2 | 7 | Fair |
| Gomot et al. (2011) | 3 | 2 | 2 | 7 | Fair |
| Goris et al. (2018) | 3 | 2 | 2 | 7 | Fair |
| Green et al. (2020) | 3 | 2 | 3 | 8 | Good |
| Haigh et al. (2023) | 4 | 1 | 2 | 7 | Good |
| Huang et al. (2018) | 3 | 2 | 2 | 7 | Fair |
| Hudac et al. (2018) | 4 | 2 | 2 | 8 | Good |
| Jansson-Verkasalo et al. (2003) | 3 | 2 | 2 | 7 | Fair |
| Jansson-Verkasalo et al. (2005) | 3 | 1 | 2 | 6 | Fair |
| Kabil et al. (2023) | 4 | 1 | 2 | 7 | Good |
| Kasai et al (2005) | 3 | 2 | 2 | 7 | Fair |
| Kemner et al. (1995) | 3 | 1 | 2 | 6 | Fair |
| Korpilahti et al. (2007) | 3 | 2 | 2 | 7 | Fair |
| Kujala et al. (2005) | 3 | 2 | 2 | 7 | Fair |
| Kujala et al. (2007) | 3 | 2 | 2 | 7 | Fair |
| Kujala et al. (2010) | 3 | 2 | 2 | 7 | Fair |
| Lassen et al. (2022) | 4 | 1 | 2 | 7 | Good |
| Lepistö et al. (2005) | 3 | 2 | 2 | 7 | Fair |
| Lepistö et al. (2006) | 3 | 2 | 2 | 7 | Fair |
| Lepistö et al. (2007) | 3 | 2 | 2 | 7 | Fair |
| Lepistö et al. (2008) | 3 | 1 | 2 | 6 | Fair |
| Lepistö et al. (2009) | 3 | 2 | 2 | 7 | Fair |
| Lindström et al. (2016) | 3 | 2 | 2 | 7 | Fair |
| Lindström et al. (2018) | 3 | 2 | 2 | 7 | Fair |
| Lortie et al. (2017) | 3 | 1 | 2 | 6 | Fair |
| Ludlow et al. (2014) | 3 | 2 | 2 | 7 | Fair |
| Mamashli et al. (2017) | 3 | 2 | 2 | 7 | Fair |
| Matsuba et al. (2024) | 3 | 1 | 2 | 6 | Satisfactory |
| Matsuzaki et al. (2017) | 3 | 1 | 2 | 6 | Fair |
| Matsuzaki et al. (2019) | 3 | 2 | 2 | 7 | Fair |
| Oram Cardy et al. (2005) | 3 | 1 | 2 | 6 | Fair |
| Randeniya et al. (2022) | 4 | 2 | 3 | 9 | Good |
| Roberts et al. (2011) | 4 | 1 | 2 | 7 | Fair |
| Ruiz-Martínez et al. (2020) | 3 | 1 | 2 | 6 | Fair |
| Sanglakh Ghoochan Atigh et al. (2024) | 4 | 1 | 2 | 7 | Good |
| Sato et a. (2025) | 3 | 1 | 2 | 6 | Satisfactory |
| Sawada et al. (2008) | 2 | na | 3 | 5 | Fair |
| Schall et al. (2024) | 4 | 1 | 2 | 7 | Good |
| Sokhadze et al. (2016) | 3 | 1 | 2 | 6 | Fair |
| Tecchio et al. (2003) | 3 | 2 | 2 | 7 | Fair |
| Vlaskamp et al. (2017) | 3 | 2 | 2 | 7 | Fair |
| Weismüller et al. (2015) | 3 | 1 | 2 | 6 | Fair |
| Yu et al. (2015) | 3 | 1 | 2 | 6 | Fair |
| Zhang et al. (2018) | 3 | 2 | 2 | 7 | Fair |
| Zhang et al. (2019) | 4 | 2 | 2 | 8 | Good |
| **na**, not applicable | | | | | |
|  |  |  |  |  |  |
